# Gene Function Analysis in the Ubiquitous Human Commensal and Pathogen *Malassezia* Genus

**DOI:** 10.1128/mBio.01853-16

**Published:** 2016-11-29

**Authors:** Giuseppe Ianiri, Anna F. Averette, Joanne M. Kingsbury, Joseph Heitman, Alexander Idnurm

**Affiliations:** a Department of Molecular Genetics and Microbiology, Duke University Medical Center, Durham, North Carolina, USA; b The Institute of Environmental Science and Research, Christchurch, New Zealand; c School of BioSciences, University of Melbourne, Victoria, Australia

## Abstract

The genus *Malassezia* includes 14 species that are found on the skin of humans and animals and are associated with a number of diseases. Recent genome sequencing projects have defined the gene content of all 14 species; however, to date, genetic manipulation has not been possible for any species within this genus. Here, we develop and then optimize molecular tools for the transformation of *Malassezia furfur* and *Malassezia sympodialis* using *Agrobacterium tumefaciens* delivery of transfer DNA (T-DNA) molecules. These T-DNAs can insert randomly into the genome. In the case of *M. furfur*, targeted gene replacements were also achieved via homologous recombination, enabling deletion of the *ADE2* gene for purine biosynthesis and of the *LAC2* gene predicted to be involved in melanin biosynthesis. Hence, the introduction of exogenous DNA and direct gene manipulation are feasible in *Malassezia* species.

## IMPORTANCE

Species in the genus *Malassezia* are a defining component of the microbiome of the surface of mammals. They are also associated with a wide range of skin disease symptoms. Many species are difficult to culture *in vitro*, and although genome sequences are available for the species in this genus, it has not been possible to assess gene function to date. In this study, we pursued a series of possible transformation methods and identified one that allows the introduction of DNA into two species of *Malassezia*, including the ability to make targeted integrations into the genome such that genes can be deleted. This research opens a new direction in terms of now being able to analyze gene functions in this little understood genus. These tools will contribute to define the mechanisms that lead to the commensalism and pathogenicity in this group of obligate fungi that are predominant on the skin of mammals.

## INTRODUCTION

*Malassezia* is a monophyletic genus of yeasts that are naturally found on human and animal skin and hair. They are also associated with a variety of clinical skin disorders, such as dandruff, atopic eczema, dermatitis, pityriasis versicolor, seborrheic dermatitis, and folliculitis; occasionally in immunocompromised hosts or patients on total parenteral nutrition, *Malassezia* can also cause systemic disease (1–3). There are 14 characterized species in this fungal genus, which differ in preferred host environment, nutritional requirements, and disease association (4). However, new species are still being described, and DNA sequence analysis from environmental samples provides evidence for other uncultured lineages, indicating that the diversity in this genus is not yet fully understood (5–7). The exact role of the fungus in human disorders still remains to be resolved, as *Malassezia* species are ubiquitous and major components of the human mycobiome on skin (8). A metagenomics approach revealed that the species most commonly found on human skin are *M. restricta*, *M. globosa*, and *M. sympodialis*; conversely, *M. furfur* is rarely found as a commensal, but it is reported as the main fungemia-causing species (9). One notable property is that all of the species of *Malassezia* are fatty acid auxotrophs, having lost their fatty acid synthetase genes; thus, they require lipids for growth.

The *Malassezia* species belong in the *Ustilaginomycotina* within the *Basidiomycota* phylum (10), which includes another human pathogen, *Cryptococcus neoformans* (*Agaricomycotina*). However, the evolutionary trajectory toward becoming pathogenic must have differed, because *Malassezia* is more closely related to the smut plant pathogens like *Ustilago maydis*, while *C. neoformans* is related to fungal saprophytes (11).

Genomic and transcriptomic studies are essential starting points to understand the biology of pathogenic fungi, the mechanisms of pathogenesis, the interaction with the host, the discovery of new targets for antifungal development, and their evolution. Genomic studies of *Malassezia* have been conducted over the last decade. In 2007, the first genome sequence for the dandruff-associated species *M. globosa* was published (12), followed in 2013 by the genome of *M. sympodialis* (13) and that of *M. pachydermatis* in 2015 (14). Most recently, Wu and colleagues sequenced, assembled, and annotated genomes for all 14 known *Malassezia* species, including multiple strains of the most widely studied species (*M. furfur*, *M. globosa*, *M. sympodialis*, and *M. restricta*), for a total of 24 genomes sequenced (9). *Malassezia* species are characterized by small genomes (from ~7 to ~9 Mbp), about half of the size of other known basidiomycete yeasts, with some species having less than 4,000 predicted genes; this is likely the result of their evolution to occupy a very limited ecological niche. Exceptions within the genus are represented by a subset of strains of *M. furfur* that have double the size of other *M. furfur* genomes, indicating genome duplication, hybridization events, or diploidy (9). Despite these extensive genome sequence resources, further work on *Malassezia* species is hampered by the current inability to modify their genes.

Genetic transformation in fungi is a key tool for studying gene function. It can be achieved through the use of lithium acetate (LiAc) and polyethylene glycol (PEG), electroporation of intact cells or protoplasts, or biolistic bombardment or mediated by the naturally conjugative bacterium *Agrobacterium tumefaciens*. Each method presents advantages and disadvantages that can differ depending on the fungal species to be transformed. Briefly, LiAc/PEG is a facile method that is routinely used for transformation of *Saccharomyces cerevisiae* and other ascomycetes, such as *Schizosaccharomyces pombe* and *Candida albicans*. Electroporation is largely used for transformation of filamentous fungi with the aim of generating targeted gene replacement mutants, and for example it is the most effective transformation method for *Mucor circinelloides* (15). However, it is less commonly used for random insertional mutagenesis due to the high proportion of unstable transformants generated and because it can cause large genomic deletions and rearrangements. Biolistic approaches are effective in transforming several filamentous fungi and yeasts, even though the biolistic approach is not the method of choice where there are alternative protocols because of the high cost of the biolistic apparatus. Biolistic transformation can be effective for insertional mutagenesis, although the transformants often show instability of the exogenous DNA. It is the standard method for the generation of targeted gene replacement mutants in the *C. neoformans*/*C. gattii* species complex. *A. tumefaciens*-mediated transformation (AMT) is an effective system for delivery of exogenous DNA in most yeasts and filamentous fungi: over 130 fungal species have been successfully transformed using AMT. It is an ideal tool for forward genetic studies due to the high transformation efficiency and single ectopic insertions; also, it does not require expensive laboratory equipment. In ascomycete fungi, AMT can also be used also for targeted gene replacement, although this cannot be achieved in some basidiomycete species, such as the *C. neoformans* and *C. gattii* complex species (15–17).

In the following study, we tested these techniques for their effectiveness in transforming two species within the *Malassezia* genus. We generated plasmids based on *Malassezia*-specific gene markers and demonstrated that AMT is a reliable and effective system to transform the two species tested, *M. furfur* and *M. sympodialis*. Importantly, we show that AMT is also effective in generating targeted gene replacement mutants.

## RESULTS

### Identification of media for the selection of *M. furfur* and *M. sympodialis* transformants.

**FIG 1:**
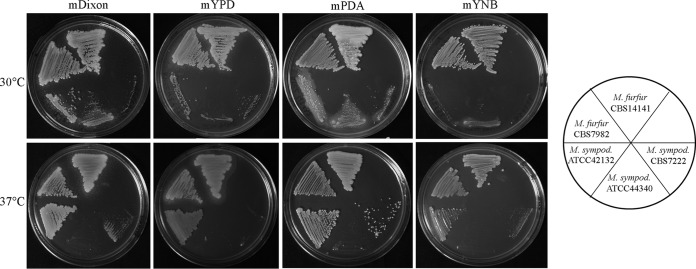
Growth of *M. furfur* strains CBS 14141 and CBS 7982 and *M. sympodialis* (*M. sympod*.) strains ATCC 42132, ATCC 44340, and CBS 7222 on four types of media (mDixon, mYPD, mPDA, and mYNB) after 5 days of incubation at 30 and 37°C.

The growth at 30 and 37°C of *M. furfur* strains CBS 14141 and CBS 7982 and *M. sympodialis* strains ATCC 42132, ATCC 44340, and CBS 7222 was tested on modified (m-) versions of the rich media yeast extract-peptone-dextrose (mYPD), Dixon’s medium (mDixon), and potato dextrose agar (mPDA), to identify the most suitable substrate for each species. In addition, growth was evaluated on nutrient-limited medium yeast nitrogen base (mYNB) to test its efficacy as a selection for potential generation of auxotrophic strains. The “m-” designation indicates a modification from the standard medium components by the inclusion of two sources of fatty acids (Tween 20 and Tween 60) and bile salts. At 30°C, the two *M. furfur* strains showed better growth in all types of media compared with the *M. sympodialis* strains. Moreover, *M. furfur* strain CBS 14141 grew better on mPDA and strain CBS 7982 on mDixon, and thus these two media were used routinely to cultivate these species. The *M. furfur* strains also had robust growth on mYNB; thus this medium is suitable for identification and characterization of auxotrophic strains. Conversely, the *M. sympodialis* strains ATCC 42132 and ATCC 44340 showed the best growth on mDixon, CBS 7222 grew best on mPDA, and all three had limited growth on minimal media at 30°C. At 37°C, *M. sympodialis* ATCC 42132 grew as well as the *M. furfur* strains in all medium types, while CBS 7222 showed no growth on mYPD and limited growth on the three remaining media, and strain ATCC 44340 was unable to grow on any medium tested at 37°C (Fig. 1).

### Development of selectable markers and transformation systems for *M. furfur* and *M. sympodialis.*

Transformation first requires the identification of selectable marker genes and regulatory regions that allow the expression of the introduced DNA in the host cells, followed by the construction of vectors containing this DNA. We previously developed transformation protocols for yeasts in the subdivision *Pucciniomycotina* (18, 19) and here followed the same strategies for the transformation of *Malassezia* species. Initially we attempted to isolate auxotrophs in the uracil biosynthesis pathway by selection on modified medium containing 5-fluoroorotic acid (m5-FOA): this approach was not possible due to the selection of *M. furfur* strains that were resistant to 5-FOA but were still able to grow in the absence of uracil (data not shown).

Therefore, the feasibility of using dominant drug markers was tested. The genes chosen were those conferring resistance to nourseothricin (*NAT*) and Geneticin/G418 (*NEO*), which have been used for the transformation of basidiomycete fungi, including the human pathogen *C. neoformans* (20) and plant pathogen *U. maydis* (21). The regulatory regions of *M. sympodialis* ATCC 42132 were chosen to drive expression of these genes due to the ease of cultivability of this strain and the availability of genome sequence (13). While the genome sequence of *M. globosa* CBS 7966 was also available (12), this species was difficult to grow. The promoter and terminator of the actin-encoding *ACT1* gene of *M. sympodialis* were amplified by PCR and fused by overlap PCR with the *NAT* and *NEO* marker PCR products. The *NAT* and *NEO* cassettes were cloned into the TOPO pCR2.1 vector and used initially for transformation attempts of *M. furfur* strains. In addition to standard protocols used successfully for the transformation of other fungi, several modifications were also performed in order to account for *Malassezia*’s unique growth conditions and requirements. While no drug-resistant colonies were obtained following transformation by lithium acetate/PEG or electroporation, several drug-resistant colonies were isolated using biolistic transformation; however, the presence of the *NEO* or *NAT* marker could not be verified by PCR analysis.

The *NAT* and *NEO* cassettes were cloned within the T-DNA region of the binary vector pPZP-201BK to generate plasmids pAIM2 and pAIM6, respectively (see Fig. S1 in the supplemental material). *Agrobacterium*-mediated transformation was carried out on strains of *M. furfur* and *M. sympodialis*. The first transformation experiment was performed using conditions reported for *Pucciniomycotina* (18), except that the induction medium contained Tween and bile salts (mIM), and yielded two stable nourseothricin-resistant (NAT^R^) colonies for *M. furfur* CBS 14141, but no transformants for the other strains. Despite the poor transformation efficiency, PCR and Southern blot analyses confirmed integration of the *NAT* marker into the CBS 14141 genome (data not shown), rather than emergence of spontaneous drug resistance.

**FIG 2:**
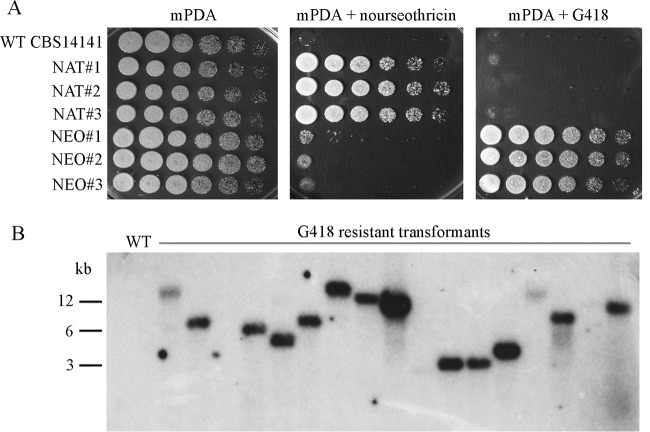
Phenotypic and genotypic characterization of *M. furfur* random insertion transformants. (A) Ten-fold serial dilutions of cellular suspensions of the *M. furfur* wild-type strain CBS 14141 and three randomly selected NAT^R^ and G418^R^ transformants spotted in a volume of 1.5 µl to nonselective mPDA medium and selective mPDA plus nourseothricin or G418 medium. (B) Southern blot of DNA from the wild-type strain CBS 14141 and 17 randomly selected G418^R^ transformants probed with the *NEO* gene. Genomic DNA was digested with XhoI, which does not cleave within the T-DNA region.

Because CBS 14141 was the only strain transformed, it was used as a model to test conditions that could enhance transformation efficiency. The best results were achieved when concentrated cultures of *Malassezia* (i.e., optical density at 600 nm [OD_600_] of ~1) were used in combination with *Agrobacterium* cultures (OD_600_ of ~1), and the coincubation step was performed on nylon membranes placed on the mIM for 3 to 6 days before being transferred onto selective medium. Under these conditions, ~10 to 100 drug-resistant transformants per coincubation plate were obtained. Fig. S2 in the supplemental material is a representative example of selective medium plates obtained following biolistic transformation and AMT. In Fig. 2A, the specific drug resistance of three NAT^R^ and G418-resistant (G418^R^) randomly selected transformants was compared with that of the wild-type strain. Southern blot analysis was performed on 17 randomly selected *M. furfur* CBS 14141 transformants obtained using the pAIM6 (*NEO*) vector (Fig. 2B). A single hybridization band that differed in size was observed for transformants, indicating a single, random T-DNA integration event into the genome of each strain; in three cases, the hybridization bands were only weakly detected, and for the wild type, no signal was observed as expected.

**FIG 3:**
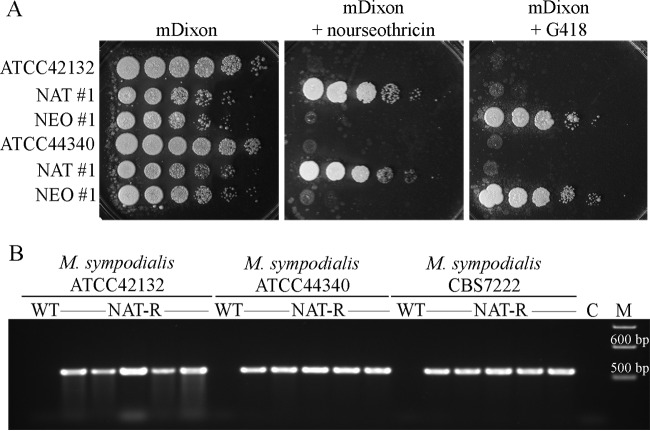
Phenotypic and genotypic characterization of *M. sympodialis* random insertion transformants. (A) Ten-fold serial dilutions of cellular suspensions of the *M. sympodialis* wild-type strains ATCC 42132 and ATCC 44340 and two randomly selected NAT^R^ and G418^R^ transformants spotted in a volume of 1.5 µl on nonselective medium mDixon and on selective mDixon plus nourseothricin or G418 medium. (B) Confirmation PCR analysis performed with primers specific for the *NAT* ORF (ai036-ai037) and DNA isolated from the *M. sympodialis* wild-type strains ATCC 42132, ATCC 44340, and CBS 7222 and five randomly selected NAT^R^ transformants derived from each parental strain. C, water control; M, marker.

Using the optimized conditions described above, AMT experiments were also carried out for *M. furfur* CBS 7982 and the three strains of *M. sympodialis*. Surprisingly, no transformants of *M. furfur* CBS 7982 were obtained following 3 or 6 days of coincubation, despite this strain growing at a similar rate to CBS 14141. Conversely, NAT^R^ colonies were obtained for all three strains of *M. sympodialis*, although the transformation efficiency was much lower than that observed for *M. furfur* CBS 14141, with a maximum of ~10 colonies per transformation plate achieved for 6 days of coincubation. Figure 3A depicts the specific drug resistance of two randomly isolated, representative NAT^R^ and G418^R^ transformants of ATCC 42132 and ATCC 44340 on selective media. Of note, when the *NEO* marker was used for *M. sympodialis* transformation, a lower transformation efficiency was obtained compared with the *NAT* marker, and when longer coincubation times were used (6 days), the emergence of spontaneous drug-resistant colonies was observed for the strains ATCC 44340 and CBS 7222 (data not shown). Figure 3B shows a representative PCR analysis of five randomly selected NAT^R^ transformants obtained for ATCC 42132, ATCC 44340, and CBS 7222. An amplicon of the expected size (576 bp) corresponding to the *NAT* open reading frame (ORF) was obtained from DNA extracted from the transformants, but not from DNA from the wild type, indicating the presence of the exogenous marker in the genomes of these three strains of *M. sympodialis*, as confirmed also by Southern blot analysis (data not shown).

### Forward genetics via *Agrobacterium tumefaciens*-mediated transformation.

**FIG 4:**
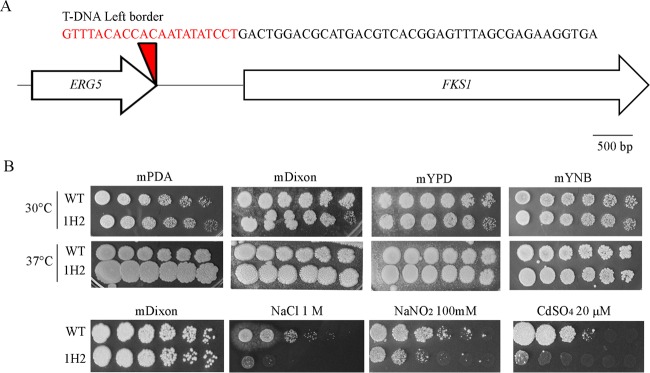
Analysis of the NAT^R^ transformant 1H2 derived from *M. furfur* CBS 14141. (A) The T-DNA–genomic DNA junction was identified through inverse PCR, depicting the sequence of the left border (red) and that of the genomic DNA of *M. furfur* CBS 14141 (red); the position of the left border in the *ERG5* gene is represented by the red triangle. (B) Ten-fold serial dilutions of cell culture of the wild-type strain CBS 14141 and transformant 1H2 on mPDA, mDixon, mYPD, and mYNB after 7 days of incubation at 30 and 37°C and on mDixon supplemented with 1.5 M NaCl, 100 mM NaNO_2_, and 20 µM CdSO_4_. Note the larger size of the colonies of strain 1H2 on mPDA at 37°C.

As proof of principle for forward genetics, the phenotypes of 96 NAT^R^ and G418^R^ transformants generated during the optimization of the AMT procedure were evaluated at 37°C and on two minimal media [mYNB and mMM (minimal medium)]. One mutant (strain 1H2) was selected that showed more pronounced growth on mPDA medium at 37°C with colonies becoming larger than wild type, and the gene that was mutated by the T-DNA insertion was identified by inverse PCR. While the right border could not be identified, the left border of the T-DNA was inserted in the last exon of the *ERG5* ortholog. In yeast *ERG5* encodes C-22 sterol desaturase, a cytochrome P-450 enzyme that catalyzes the formation of the C-22,23 double bond in the sterol side chain in ergosterol biosynthesis (22). The gene adjacent to *ERG5* in the CBS 14141 genome is *FKS1* (Fig. 4A), which encodes an essential catalytic subunit of 1,3-β-d-glucan synthase involved in cell wall synthesis and maintenance, and it is the target of the echinocandin drugs (23). Strain 1H2 was tested for its sensitivity to a number of stress conditions, and besides the more pronounced growth observed on mPDA medium at 37°C, it also showed an increased sensitivity to the osmotic and nitrosative stress-inducing agents sodium chloride (NaCl) and sodium nitrite (NaNO_2_), respectively, and to the heavy metal cadmium sulfate (CdSO_4_) (Fig. 4B). Surprisingly, strain 1H2 did not show any sensitivity to drugs that are expected to target either the *ERG5* or *FKS1* gene (i.e., fluconazole, amphotericin B, and caspofungin) and to chemicals or conditions that resulted in clear phenotypic alteration of the yeast *erg5* mutant (SDS, Congo red, geldanamycin, cycloheximide, benomyl, hydroxyurea, sirolimus, FK506, cyclosporine, or UV stress) (data not shown).

### Targeted mutagenesis via *Agrobacterium tumefaciens*-mediated transformation.

To test if AMT could be suitable for targeted gene replacement by homologous recombination in *Malassezia*, two genes were selected as they would allow for facile phenotypic identification of strains with the genes mutated. *ADE2* encodes phosphoribosylaminoimidazole carboxylase, which catalyzes a step in the “*de novo*” purine nucleotide biosynthetic pathway; *ade2* mutants of *S. cerevisiae* and other fungi are red, and thus they have long served as markers for transformation and genetic experiments (24). The *LAC2* gene was chosen based on the production of melanin from the substrate l-3,4-dihydroxyphenylalanine (l-DOPA) by the strain NBRC 0656 of *M. furfur* (25). Based on what is known in fungi, including the human pathogen *C. neoformans*, laccase-deficient mutants have colony pigmentation that ranges from white to brown due to the inability to accumulate the dark pigment melanin on l-DOPA medium, thus enabling discrimination from wild-type cells (26).

A draft version of the genome of *M. furfur* CBS 14141 (now available in NCBI [9]) was analyzed using tBLASTn with the *S. cerevisiae* Ade2 protein as the query, and one copy of the gene was identified and the sequence retrieved, including an additional 2,000 bp of 5′ and 3′ flanking nucleotides. Laccase genes were searched using tBLASTn queries with Lac1 (encoded by CNAG_03465 [*LAC1*]) and Lac2 (encoded by CNAG_03464 [*LAC2*]) of *C. neoformans* strain H99. The closest orthologs of both *C. neoformans* Lac1 and Lac2 in CBS 14141 comprised the same gene (E values of 3E−59 and 6E−60, respectively), and bidirectional BLAST confirmed that this gene is the ortholog of *C. neoformans LAC2*, which is the designation that we use in this article. The second best hit of CBS 14141 was an ortholog of the *C. neoformans* gene CNAG_06241, which encodes an acidic laccase.

**FIG 5:**
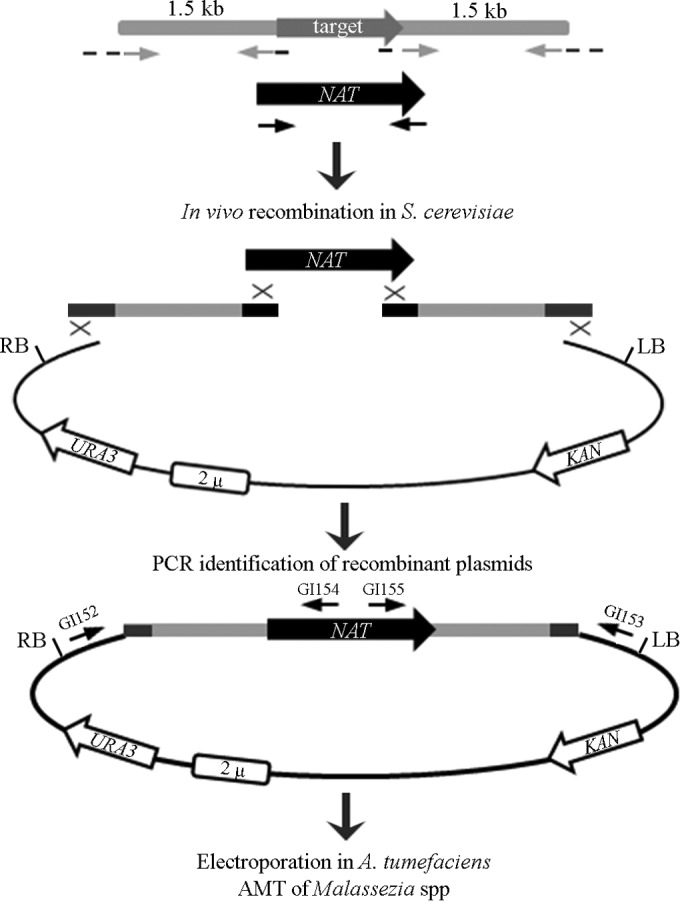
Strategy used for the generation of binary vectors used in targeted gene replacement experiments. Regions (1.5 kb) flanking the target genes were amplified by PCR using primers that contain additional sequences for recombination with pGI3 (dashed lines) and with the *NAT* cassette (black lines). The 5′ and 3′ flanking regions and the *NAT* cassette were fused together within the T-DNA region of the pGI3 plasmid backbone by *in vivo* recombination in *S. cerevisiae*. Recombinant plasmids were identified from *S. cerevisiae* transformants by PCR and electroporated into *A. tumefaciens* strain EHA105 for subsequent AMT experiments.

To generate vectors for *A. tumefaciens*-mediated targeted mutagenesis of *ADE2* and *LAC2*, DNA fragments were assembled using recombination within *S. cerevisiae* into the plasmid pGI3 (48). The strategy to assemble simultaneously and clone the gene replacement cassettes within the T-DNA of pGI3 is illustrated in Fig. 5. Briefly, three PCR fragments that include the *NAT* marker gene and the 1.5-kb upstream (5′) and downstream (3′) regions flanking the target genes *ADE2* and *LAC2*, as well as plasmid pGI3 digested with KpnI and BamHI (both enzymes cut within the T-DNA region), were used to transform *S. cerevisiae*; homologous regions between the PCR fragments and the digested plasmid allowed endogenous recombination events. Colony PCR was used to identify yeast transformants bearing correctly assembled plasmids, which were then extracted from yeast and introduced via electroporation into *A. tumefaciens* EHA105, and these bacterial strains were then used for AMT according to the optimized procedure described above.

**FIG 6:**
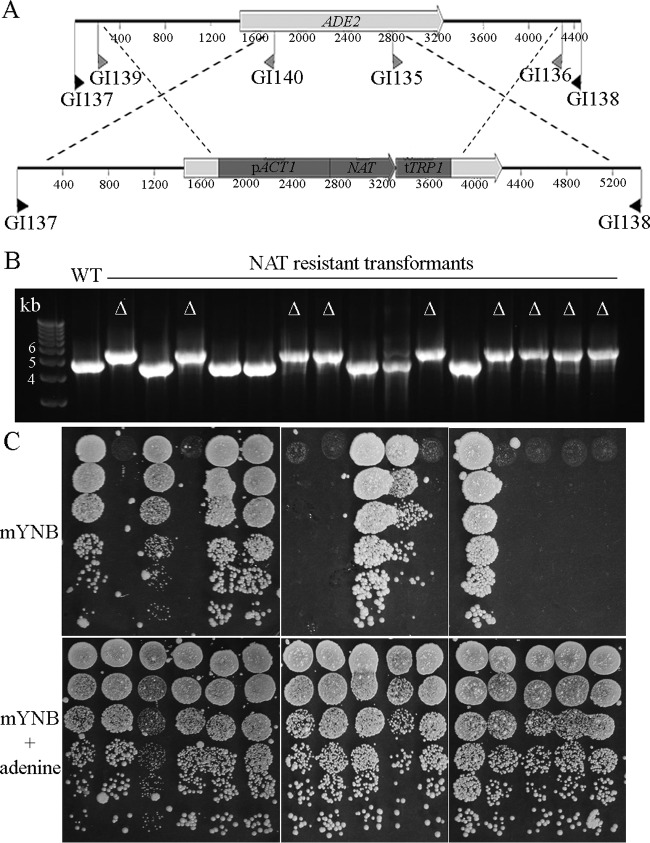
Targeted gene replacement of the *M. furfur ADE2* gene. (A) Schematic representation of the targeted gene replacement of the *ADE2* gene of *M. furfur* CBS 14141 using the *NAT* marker. Primers used to amplify the flanking regions of 1.5 kb (gray arrows), and screening primers (black arrows) used to identify *ade2*Δ alleles are shown. (B) A 5.4-kb PCR product corresponding to *ade2*::*NAT* was obtained for the *ade2*Δ mutants (indicated by the symbol Δ) compared with a 4.4-kb *ADE2* amplicon obtained for the wild-type and nonmutant NAT^R^ transformants. (C) The wild type and all of the NAT^R^ transformants obtained were 10-fold serially diluted and spotted in volumes of 1.5 µl onto mYNB and mYNB supplemented with adenine. All of the putative *ade2*Δ strains, as identified by PCR, were unable to grow on medium without adenine, confirming the expected adenine auxotrophy.

NAT^R^ transformants of *M. furfur* CBS 14141 were screened by PCR for homologous recombination events causing gene replacement. For the *ADE2* gene, the construct was designed to replace a 1,041-bp region with the 2,028-bp *NAT* marker (Fig. 6A). PCR amplification using external screening primers produced two amplicons—one of 4.5 kb for the CBS 14141 wild type and six NAT^R^ strains and another of 5.5 kb for the other nine NAT^R^ strains. This indicates that the CBS 14141 *ADE2* gene was replaced in 9 out of the 15 transformants (Fig. 6B). The strains were tested for their growth on minimal mYNB medium supplemented or not with adenine. All nine *ade2Δ* mutants identified by PCR exhibited adenine auxotrophy (Fig. 6C). Interestingly, the *M. furfur ade2Δ* mutants did not show a classical pink pigmentation as in the model yeast, but their color varied from light pink on rich media to yellow on minimal medium mYNB supplemented with adenine (data not shown).

**FIG 7:**
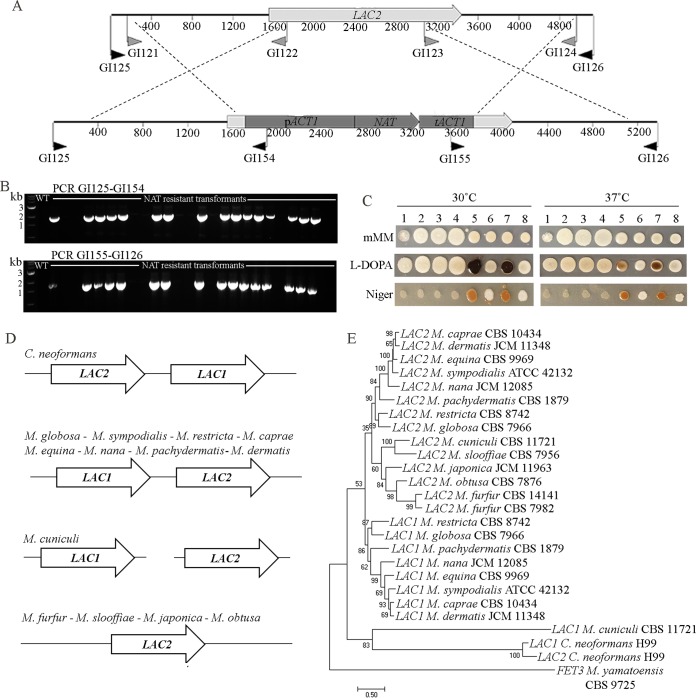
Targeted gene replacement of the *M. furfur* CBS 14141 *LAC2* gene. (A) Schematic representation of the targeted gene replacement of the *LAC2* gene of *M. furfur* CBS 14141 using the *NAT* marker. Primers used to amplify the flanking regions of 1.5 kb are shown (gray arrows) as well as screening primers (black arrows) used to identify *lac2*Δ as shown in panel B: an ~1.8-kb PCR product was obtained for the *lac2*Δ mutants compared with the null amplification obtained for the wild-type and nonmutant NAT^R^ transformants. (C) A 1.5-µl cellular suspension of the wild-type strain CBS 14141 (lane 1), two independent *lac2*Δ mutants (lanes 2 and 3), a NAT^R^ transformant obtained in the same AMT experiment (lane 4), *C. neoformans* H99 (lane 5), and the *C. neoformans lac1*Δ (lane 6), *lac2*Δ (lane 7), and *lac1*Δ *lac2*Δ (lane 8) mutants used in reference 27 were spotted on mMM, l-DOPA, and Niger seed extract media to assess melanin production; pictures were taken after 8 days of incubation at 30 and 37°C. (D) Physical genomic arrangement of the *LAC1* and *LAC2* genes of *C. neoformans* H99 and representative strains of the 14 sequenced *Malassezia* species as identified in the genome assemblies. (E) The predicted ORFs from the *LAC1* and *LAC2* genes in *Malassezia* species and *C. neoformans* were used to generate a phylogenetic tree using the maximum likelihood method.

For the targeted replacement of the *LAC2* gene, 25 NAT^R^ candidates were screened by PCR using primers designed beyond the regions of DNA used in the deletion allele in combination with internal *NAT* primers (Fig. 7A). For 16 transformants, specific amplicons of ~1.8 kb for both the 5′ and 3′ sides were obtained that are diagnostic of correct gene replacement (Fig. 7A and B). Phenotypically, there were no growth or pigmentation differences between the *M. furfur* CBS 14141 wild-type strain, two independent *lac2*Δ mutants and an ectopic NAT^R^ transformant, on mMM supplemented or not with l-DOPA or on Niger seed agar, both at 30 and 37°C. Conversely, previously reported *C. neoformans* strains (27) used as control for the *Malassezia*-optimized melanin media showed strong melanin production by the wild-type H99 strain at 30°C, lack of melanin by the *lac1*Δ and *lac1*Δ *lac2*Δ mutants, and melanin production similar to that of the wild type by the *lac2*Δ mutant (Fig. 7C).

Laccase-encoding genes in fungi are characterized by the presence of multicopper oxidase domains and are known to be involved in iron and copper homeostasis and resistance to oxidative, genotoxic, and other stress conditions; they are also reported to play a role in virulence in fungal human pathogens (28–30). Analysis on the Conserved Domains Database (CDD) revealed the presence of three multicopper oxidase domains also in the *LAC2* gene of *M. furfur* CBS 14141. Following these findings, the growth of the *M. furfur lac2*Δ mutant was evaluated both on low- and high-iron or copper media and also in the presence of chemicals that are known to induce oxidative, nitrosative, and genotoxic stresses, such as hydrogen peroxide, nitric oxide, hydroxyurea, and cadmium sulfate. Moreover, the *M. furfur lac2*Δ mutant was further tested in the presence of the cell-wall-damaging agents NaCl, Congo red, and SDS, under UV stress and alkaline conditions and in the presence of the antifungal drugs fluconazole, amphotericin B, and caspofungin. Surprisingly, in no case were phenotypic differences between the *M. furfur lac2*Δ mutant and the wild-type strain detected, clearly indicating an unknown role of the *LAC2* gene of *M. furfur* (see Fig. S3 in the supplemental material; data not shown).

Laccase-encoding genes in the genome assemblies of representative strains for the 14 sequenced *Malassezia* species were identified using as queries the Lac1 and Lac2 proteins of *C. neoformans*, and their start and the stop codons were predicted based on the respective annotated orthologs in *M. sympodialis*, *M. globosa*, and *M. pachydermatis* (12–14) (see Fig. S4 in the supplemental material). Bidirectional BLAST hits of these *Malassezia* ORFs against the *C. neoformans* protein set found the two laccases Lac1 and Lac2, and based on this analysis, the laccase-encoding genes of *Malassezia* were named *LAC1* and *LAC2*, respectively. In eight *Malassezia* species, *LAC1* and *LAC2* were found to be adjacent in the genome but arranged in opposite directions compared to *C. neoformans*, while in *M. cuniculi*, they lie on different scaffolds in the genome assembly. For *M. furfur*, *M. slooffiae*, *M. japonica*, and *M. obtusa*, only the *LAC2* gene is present. Neither *LAC1* nor *LAC2* was found in *M. yamatoensis*, with the closest homolog to the *C. neoformans* H99 Lac1 being *FET3*, which encodes a ferro-O_2_-oxidoreductase (Fig. 7D). The phylogenetic tree of Fig. 6E shows three main groups: one that consists of the *LAC2* gene present in all of the *Malassezia* species examined, a second that includes the gene *LAC1*, and a third group that consists of *C. neoformans LAC1* and *LAC2* and a divergent *LAC1* in *M. cuniculi*; *M. yamatoensis FET3* did not group with the laccase-encoding genes (Fig. 7E).

Despite the lack of phenotypes for the *lac2*Δ mutant of *M. furfur* CBS 14141, the results obtained indicate that AMT is an efficient method of generating targeted mutants through homologous recombination in *M. furfur*.

## DISCUSSION

*Malassezia* is a genus of fungi that includes species that are the primary commensal organisms found on the exterior of animals. Apart from their normal commensal role on the skin of all people, the *Malassezia* species cover a full range of severity of human mycoses, from the relatively rare cases of systemic disease to what is likely the most common fungus-associated disorder in humans, dandruff. Loss of their fatty acid synthetase genes, thus requiring them to have a lipid source for growth, is likely one factor linking them to animal hosts. Lipid auxotrophy together with a subsequent loss of other genes associated with the commensal lifestyle, including those involved in carbohydrate metabolic processes and hydrolysis activity, results in challenges in culturing *Malassezia in vitro*. These challenges have substantially hampered efforts to better understand *Malassezia* fungi, despite their ubiquitous contribution to the human microbiome and association with clinical disorders.

Analysis of the genome sequences of the 14 *Malassezia* species known at the time revealed idiosyncrasies, such as the presence of unique genes of unknown function, genes acquired through horizontal gene transfer that are predicted to represent key gain-of-function events in the *Malassezia* genus, and the presence of several lipase families that are necessary to break down lipids for growth (9). Although this sequencing represents a valuable resource for researchers and clinicians, its utility is largely unexploited and limited mainly to bioinformatic applications, without a system that allows genetic transformation and gene manipulation.

In the present work, we report for the first time the transformation of two species of *Malassezia*, *M. sympodialis* and *M. furfur*. These two species are characterized by favorable growth under *in vitro* conditions compared with the other *Malassezia* species and also can be considered in humans as representative of the commensal and fungemia-causing groups, respectively (9). The vectors generated for transformation consisted of the *NAT* and *NEO* marker genes cloned under the control of the native promoter and terminator of the actin-encoding gene *ACT1* of *M. sympodialis* (Fig. S1), because in previous studies the use of genus-specific regulatory regions has been shown to play a crucial role enabling successful expression of heterologous genes in basidiomycetous fungi (19, 31, 32).

The most common techniques for transformation of fungi were tested for the ability to deliver the vectors designed for gene expression in *M. furfur* strains. Transformation with LiAc/PEG or electroporation methods did not yield transformants. In other studies, when these transformation techniques were unsuccessful using intact cells, they were utilized with protoplasts, which is subject to the vagaries of the ability of available cell-wall-degrading enzymes to work on the species being studied, and thus protoplast-based transformation was not tested for *M. furfur*. When biolistic transformation was tested, drug-resistant strains of *M. furfur* were obtained independently in different laboratories. However, the presence of the exogenous marker genes was not substantiated by molecular analyses. This suggested that biolistic transformation was unsuccessful, possibly due to the thick cell wall of *Malassezia* (33), or to the emergence of spontaneous drug resistance, which could be due to heteroresistance, as observed in the human pathogen *C. neoformans* (34), or multidrug resistance (MDR) through the upregulation of pumps that export drugs from the cell. Finally, the strong tendency of *M. furfur* to form numerous clumps also could have contributed to the selection of false-positive clones, although real drug-resistant transformants obtained with AMT could be clearly distinguished from the clump-forming background (Fig. S2). Indeed, researchers aiming to produce mutant strains should take this cell clumping property found in many *Malassezia* strains into consideration when aiming to purify transformants.

*A. tumefaciens*-mediated transformation (AMT) was thus the only technique successful for transformation, and it initially occurred in only one strain of *M. furfur* and at an efficiency that was low compared with those of other fungal species, wherein hundreds to thousands of transformants can be generated under the equivalent experimental conditions used for *M. furfur*. The use of membranes during coincubation that allowed closer contact between bacterial and fungal cells and a higher concentration of the *Malassezia* cells compared to the recommendations for other yeasts improved the transformation efficiency of *M. furfur* (Fig. 2) and resulted in the generation of transformants for three different strains of *M. sympodialis* (Fig. 3), indicating that the poor growth of the *Malassezia* species on the mIM may be a factor limiting the efficiency of transformation.

AMT of fungi is mainly used as a tool for functional genetic studies through forward genetics using random T-DNA insertions (17), which in *M. furfur* and *M. sympodialis* could be limited by the low transformation efficiency. Nevertheless, *Malassezia* genomes are smaller (<10 Mb) than those of many other fungi, and hence compared to other fungal systems, a smaller number of insertional mutants for forward genetic screens would be required to saturate the genome with insertions. In a proof-of-principle forward genetic screen of less than 100 transformants, one strain (transformant 1H2) bearing a T-DNA insertion in the *ERG5* gene was isolated as showing more pronounced growth on mPDA at 37°C than the wild-type strain (Fig. 4). This mutant also showed increased sensitivity to osmotic, nitrosative, and genotoxic stresses, consistent with plasma membrane defects due to alteration in the ergosterol biosynthetic pathway. However, strain 1H2 did not show altered phenotypes when tested on a number of antifungal drugs and chemicals known to affect the phenotypic response of the *erg5* mutant in yeasts and fungi (22, 35, 36). Although further experiments, such as functional complementation or targeted gene replacement, are needed to confirm if the phenotypes obtained are due to mutation of the *ERG5* gene, the small number of mutants tested and the ability to identify one junction validate the feasibility of using AMT for forward genetics.

The RNA interference pathway has been lost during evolution in all 14 *Malassezia* species characterized (9), and therefore gene targeting using RNA silencing methods cannot be carried out and necessitates that approaches like gene replacement by homologous recombination strategies be employed instead. To test if the AMT method would be suitable for targeted gene replacement by homologous recombination, the *ADE2* and *LAC2* genes were selected because they are predicted to allow facile identification of the mutated phenotype according to their function in other fungi (24, 26). Both genes were mutated in *M. furfur* with the optimized transformation conditions using flanking regions of 1.5 kb homologous to the targeted gene, a standard length for basidiomycetous fungi (17). While the *M. furfur ade2*Δ mutant showed the expected phenotype (i.e., adenine auxotrophy [Fig. 6]), the lack of a phenotype for the *lac2*Δ mutant did not clarify the function of the laccase gene in *M. furfur* (Fig. 7C; Fig. S3). Beside the lack of involvement of *LAC2* in iron and copper homeostasis and stress responses (Fig. S3), the most surprising finding was the absence of macroscopic production of melanin by *M. furfur* strain CBS 14141 on modified l-DOPA and Niger seed extract, both confirmed to act as substrates for melanization in the strain of *C. neoformans* used as a positive control (Fig. 7C) (27). Also *M. furfur* strain CBS 7982 was not able to accumulate dark melanin on those medium types (data not shown). These findings are in contrast with those of Youngchim and colleagues (25), who observed melanin synthesis by strain NBRC 0656 of *M. furfur* on l-DOPA, medium conditions which also induced the production of hypha-like forms not observed for strains CBS 14141 and CBS 7982. However, in another study, Gaitanis and colleagues showed that more than 10 strains of *M. furfur* displayed little or no melanin production on l-DOPA medium (37), indicating that strains that belong to the same species, *M. furfur*, can be characterized by relevant phenotypic differences. As regards the melanin production of *M. furfur* NBRC 0656, it seems that this strain is related to the *M. furfur* hybrid CBS 1878 (Teun Boekhout, personal communication), and hence its ability to produce melanin could be due to the combined activity of two *LAC2* genes.

Genome resources available in GenBank for the *Malassezia* genus (9) were used to search the laccase genes in representative strains of the 14 sequenced *Malassezia* species to elucidate the genetic basis of melanin biosynthesis (Fig. S4). Eight *Malassezia* species have two laccase-encoding genes adjacent in the genome, in the same orientation and sharing high similarity (>60%), mirroring the situation in *C. neoformans* (27, 30); however, evidence for gene arrangements and gene loss are also present within the *Malassezia* genus (Fig. 7D). The phylogenetic analysis (Fig. 7E) clearly shows divergence between the laccase-encoding genes of *C. neoformans* and *Malassezia* species, which supports the lack of melanization observed in our experiments. This is also corroborated by the domain analysis of the predicted Lac1 and Lac2 proteins, which in *C. neoformans* are characterized by the presence of specific diphenol oxidase domains known to have a role in melanin biosynthesis, while in *Malassezia* species, they contain uncharacterized multicopper oxidase and/or laccase-like multicopper oxidase domains with likely less specialized function. The only exception is represented by Lac1 of *M. cuniculi*, which shares high homology with multicopper oxidases of ascomycetous fungi and contains diphenol oxidase and fungal laccase domains. Therefore, based on the present analysis, none of the *Malassezia* species should have levels of melanin production comparable to those of *C. neoformans*, with the possible exception of *M. cuniculi*. Corroborating this statement, Gaitanis and colleagues (37) demonstrated for several *Malassezia* species the ability to produce melanin on l-DOPA but at lower rate than those of *C. neoformans* and *C. gattii*.

The use of AMT for targeted gene replacement in basidiomycetous fungi is not universally applicable, for example as not possible in *C. neoformans* (38). Its successful use in *M. furfur* represents the second example within this phylum along with the *Pucciniomycotina* red yeasts (19). Compared to the red yeasts, a higher efficiency of gene replacement was observed, with values of 60% (for the *ade2*::*NAT* construct) and 64% (for the *lac2*::*NAT* construct) versus the 6% and 14% obtained for *Sporobolomyces* sp. using AMT and biolistic transformation, respectively (19). Intriguingly, targeted gene replacement through AMT and a high rate of homologous recombination were recently achieved also in *M. sympodialis* (data not shown), indicating that this might be an intrinsic characteristic of the *Malassezia* genus and might be due to different mechanisms of regulation of the DNA repair pathways that mediate homologous recombination or to the absence of RNA interference components. This represents a considerable advantage that compensates for the low efficiency of AMT achieved in *M. furfur* and *M. sympodialis*.

The results presented in this project serve as a foundation for the research community to study *Malassezia* at the level of gene function. It is expected that plasmids and protocols presented in this study will be tested for their effectiveness in other *Malassezia* species and can be applied to perform larger-scale forward and reverse genetics, thereby advancing knowledge about genes within this genus of fungi. An effective transformation system allowing studies of gene function, combined with studies toward the discovery of the sexual cycle and the development of animal models under way in our laboratories, promises to better illuminate the mechanisms that lead to the commensalism and pathogenesis of species within the *Malassezia* genus.

## MATERIALS AND METHODS

### Strains and growth conditions.

The strains used in this study include the haploid strains *M. furfur* CBS 14141 (previously named JPLK23) and CBS 7982, *M. sympodialis* type strain CBS 7222, and sequenced strains ATCC 42132 and ATCC 44340 (13). *M. furfur* CBS 14141 was regularly grown on PDA modified to contain a source of fatty acid (Tween 60 [4 ml/liter], Tween 20 [1 ml/liter], and ox bile [4 g/liter]); the addition of these three compounds allowed the growth of the *M. furfur* and *M. sympodialis* strains on several common media used for the culture of other fungi, and in the text, they are referred as modified (m-) media. The *M. furfur* strain CBS 7982, and the three *M. sympodialis* strains were grown on modified Dixon’s medium (mDixon), which is the medium routinely used for culturing *Malassezia* species (9).

Attempts to isolate spontaneous uracil auxotrophs were performed according to standard yeast methods (39). In brief, the wild-type strains of *M. furfur* were streaked for single colonies, and individual colonies were inoculated for overnight (ON) growth in 2 ml of modified potato dextrose broth (mPDB). Cells were centrifuged, washed, and plated onto modified yeast nitrogen base (mYNB; Difco, Sparks, MD) medium containing 5-fluoroorotic acid (5-FOA [1 g/liter]) and uracil (20 mg/liter). 5-FOA-resistant colonies were inoculated in nonselective mPDA medium and then plated on mYNB with or without 5-FOA.

### Generation of plasmids for *Malassezia* transformation.

The DNA regions corresponding to the promoter and terminator of the actin-encoding gene *ACT1* of *M. sympodialis* strain ATCC 42132 were retrieved through BLASTx analysis using the *C. neoformans ACT1* gene as a query. PCR amplifications were performed using *Ex Taq* (TaKaRa, Shiga, Japan). For the p*ACT1-NAT-*t*ACT1* construct, *NAT* was amplified with primers ai036-ai037 from plasmid pPZP-NATcc. The promoter was amplified with primers ALID2078-ALID2019 and terminator with primers ALID2080-ALID2081 from *M. sympodialis* DNA. The overlap PCR used primers ALID2078-ALID2081. For the *P_ACT1_-NEO-T_ACT1_* construct, *NEO* was amplified with primers ALID2141-ALID2142 from plasmid pPZP-NEO11. The promoter was amplified with primers ALID2078-ALID2139 and the terminator with primers ALID2081-ALID22140 from *M. sympodialis* DNA. The overlap PCR used primers ALID2078-ALID2081. The primers used in this study are listed in Table S1 in the supplemental material. The PCR products were purified from 0.8% agarose gels, and cloned into the pCR2.1 TOPO vector (Invitrogen, Carlsbad, CA). Inserts that did not contain errors associated with PCR amplification were identified by DNA sequencing. The plasmids containing the *NAT* or *NEO* genes were named pAIM1 and pAIM5, respectively. The gene markers were excised from these plasmids using EcoRI digestion and ligated using T4 DNA ligase (New England Biolabs, Ipswich, MA) into the EcoRI site of plasmid pPZP-201BK, which is a plasmid backbone for *Agrobacterium tumefaciens* transformation (40). Chemically competent *Escherichia coli* cells were transformed with ligated fragments, and the transformants were selected on medium consisting of Luria-Bertani (LB) agar plus kanamycin (50 mg/liter). A single clone for each gene was chosen (pAIM2 and pAIM7). The selected constructs were introduced into *A. tumefaciens* strain EHA105 by electroporation, and transformants were selected on LB agar containing kanamycin (50 mg/liter).

### Transformation of *Malassezia furfur* and *M. sympodialis.*

The transformation techniques commonly used in fungi were initially tested for the two haploid strains of *M. furfur* that were chosen due to their ability to grow more vigorously than *M. sympodialis* strains. *M. furfur* CBS 14141 and CBS 7982 cells for transformation were obtained by ON growth in mPDB and liquid mDixon, respectively. Plasmids pAIM1 and pAIM5 were used for transformation with lithium acetate and polyethylene glycol (PEG), electroporation, and biolistic analysis.

Transformation mediated by lithium acetate and PEG used for the model yeasts *Saccharomyces cerevisiae*, *Schizosaccharomyces pombe*, and other ascomycetous fungi was performed as reported by Ito et al. (41). Briefly, ON cultures were washed once with 0.1 M lithium acetate, and about 1 µg of plasmid was added to the cellular suspension in the presence of 40% PEG 3750. Reaction mixtures were incubated at 30°C for 30 min and then heat shocked in a water bath for 15 min at 42°C and plated onto selective media, which included mPDA plus nourseothricin (100 µg/ml) or G418 (100 µg/ml) for CBS 14141, and mDixon plus nourseothricin (100 µg/ml) or G418 (100 µg/ml) for CBS 7982.

For electroporation experiments, cells were treated according to the procedure developed for *C. neoformans* (42). Briefly, cells cultured overnight were washed twice with double-distilled water (ddH_2_O) and resuspended in 50 ml ice-cold electroporation buffer (EB; 10 mM Tris-HCl [pH 7.5], 1 mM MgCl_2_, 270 mM sucrose) containing dithiothreitol (DTT; 1 mM). Cells were incubated on ice for 10 min and then pelleted and resuspended in 300 µl of ice-cold EB buffer without DTT. About 1 µg of pAIM1 and pAIM5 were electroporated using prechilled 2-mm-gap cuvettes into CBS 14141 cells using 6 different conditions used for fungi as reported in the Bio-Rad Gene Pulser Xcell manufacturer’s instructions: 1,500 V (voltage), 25 µF (capacitance), and 200 ohms (resistance); 2,000 V, 25 µF, and 200 ohms; 2,300 V, 25 µF, and 200 ohms; 500 V, 50 µF, and 300 ohms; 1,000 V, 50 µF, and 150 ohms; and square wave, 1,000 V, 1.0-ms pulse length, and 2 pulses with a 5-s pulse interval. Cells were plated on selective media as indicated above.

Biolistic experiments were initially performed as for *C. neoformans* (43). Briefly, transformation was directly on mPDA plus 0.5 M sorbitol, followed by 3 h of recovery at room temperature and transfer to the selective mPDA plus nourseothricin or G418 (100 µg/ml) medium. The sorbitol concentration was decreased to 0.5 M due to the inability of *M. furfur* strains to grow on 1 M. Subsequently, other conditions were tested. Overnight cultures of *M. furfur* cells were (i) plated on selective media and transformed with no recovery time, (ii) washed with EB, plated on selective media, and then transformed with no recovery time, (iii) plated on mPDA agar medium plus 0.5 M sucrose, allowed to grow for 3 h or overnight, transformed, allowed to recover for 3 h or overnight, and then transferred on selective media, or (iv) washed with EB, plated on mPDA agar medium plus 0.5 M sucrose, allowed to grow for 3 h or overnight, transformed, allowed to recover for 3 h or overnight, and then transferred on selective media.

*A. tumefaciens*-mediated transformation was initially performed following protocols developed for other basidiomycete yeasts (18, 20), with the only modification being the use of a modified induction medium (mIM containing 100 µM acetosyringone [44]) that allowed the growth of *M. furfur* and *M. sympodialis*. This protocol yielded a very low efficiency of transformation only for *M. furfur* CBS 14141, and it was not effective for *M. sympodialis*. Optimization steps for transformation included (i) the use of concentrated fungal cellular suspensions, (ii) the use of sterile nylon membranes placed on mIM during coincubation coupled with the generation of slightly concave surfaces using a sterile pestle to facilitate cell-to-cell contact and to avoid the liquid running across the plate surfaces that have altered properties due to their Tween content, and (iii) longer coincubation periods up to 6 days. As improvements, *M. furfur* and *M. sympodialis* were grown for 2 days at 30°C until the cultures reached an OD_600_ of ~1, and the engineered *A. tumefaciens* strain was grown overnight and then diluted to an OD_600_ of ~1. Four mixtures of these fungal and bacterial cellular suspensions at the indicated OD values or diluted further to 1/10 concentration were spotted directly onto the concave surface of the nylon membranes placed on mIM agar. These were coincubated for 3 to 6 days at room temperature (plates maintained without Parafilm) prior to transfer of the dual cultures to mPDA (for *M. furfur* CBS 14141) or mDixon (for *M. furfur* CBS 7982 and *M. sympodialis* strains) supplemented with nourseothricin or G418 (both at 100 µg/ml) to select for fungal transformants, and cefotaxime (200 µg/ml) to inhibit *Agrobacterium* growth.

### Targeted gene replacement in *M. furfur* strain CBS 14141.

Plasmids for delivering the gene disruption cassettes were generated by *in vivo* recombination in *Saccharomyces cerevisiae* using the binary vector pGI3, which contains features allowing its replication in *E. coli*, *A. tumefaciens* and, *S. cerevisiae* (48).

The *M. furfur ADE2* gene was identified through tBLASTn analysis using as a query the *S. cerevisiae* Ade2 protein. The 5′ and 3′ regions flanking the target *ADE2* gene were amplified from genomic DNA of CBS 14141 using primers GI139-GI140 and GI136-GI137, respectively; these primers also have homologous regions for recombination within the T-DNA of pGI3. Similarly, the *M. furfur LAC2* gene was identified through tBLASTn in the genome assembly using the Lac1 and Lac2 proteins of *C. neoformans* as the query. The 5′ and 3′ flanking regions were amplified with primers GI121-GI122 and GI123-GI124, respectively, which contain homologous regions for recombination. The *NAT* gene used as a selectable marker was amplified from plasmid pAIM1 using primers ALID2078-ALID2081. Approximately 1 μg of pGI3 was digested with KpnI and BamHI and introduced into *S. cerevisiae*, together with the 5′ and 3′ *ADE2* or *LAC2* flanking regions and the *NAT* marker.

To assess correct recombination for the newly generated plasmids, single colonies of *S. cerevisiae* transformants were screened by PCR using primers homologous to regions outside of the plasmid pGI3 involved in the recombination event (GI152-GI153) in combination with primers specific for the *NAT* marker (GI154-GI155). Positive clones were grown ON in YPD and subjected to phenol-chloroform-isoamyl alcohol (25:24:1) plasmid extraction using the protocol reported by Hoffman (45). The obtained DNA was then introduced into the *A. tumefaciens* EHA105 strain by electroporation, and the transformants were selected on LB plus 50 µg/ml kanamycin.

Targeted gene replacements in *M. furfur* CBS 14141 were performed using *Agrobacterium*-mediated transformations using the optimized protocol reported above. Transformants were purified to single colonies and verified by PCR; for identification of the *ade2*Δ mutant, primers GI137-GI138 were used, and for identification of the *lac2*Δ mutant, primers GI125-GI154 and GI126-GI155 were used. Primer sequences are listed in Table S1.

### Nucleic acid manipulation.

Genomic DNA was isolated using a CTAB extraction buffer protocol (46). For PCR, *Ex Taq* (TaKaRa, Otsu, Japan) was used according to the manufacturer’s instructions to amplify the ORF of the *NAT* gene with primers ai036-ai037 from randomly selected transformants; PCR conditions were 30 s at 94°C, 30 s at 59°C, and 45 s at 72°C for 33 cycles, with an initial denaturation of 2 min at 94°C and a final extension of 5 min at 72°C.

For Southern blot analysis, DNA was digested with XhoI, resolved on 0.8% agarose–1× Tris-acetate-EDTA (TAE) gels, and blotted to Zeta-Probe membranes (Bio-Rad, Hercules, CA). Membranes were hybridized with fragments of the *NEO* gene amplified from pAIM5 using primers ALID2078-ALID2081, and radiolabeled with [α-^32^P]dCTP using the prime-it II random primer labeling kit according to manufacturer’s instructions (Agilent Technologies, Santa Clara, CA).

The inverse-PCR technique was employed to determine the DNA sequences that flanked the T-DNA insertion in strain 1H2 as previously described (18). Briefly, ~2 µg of genomic DNA was digested with an individual restriction enzyme, the DNA was purified through a Qiagen column, and 8.5 µl was ligated with T4 DNA ligase in a 10-µl total volume overnight at 4°C. The ligation was used directly as the template in PCR with primers ai076 and ai077, M13F and ai076, or M13R and ai077. LA *Taq* (TaKaRa, Otsu, Japan) with GC buffer I was used according to the manufacturer’s instruction. The PCR product was resolved on agarose gels, excised, purified, and sequenced with primers ai076 and ai077. The DNA sequences were searched by tBLASTx analysis against the non-annotated assembly of *M. furfur* CBS 14141, and the DNA sequence retrieved was then subjected to BLASTx against several databases (*M. sympodialis*, GenBank, and SGD [*Saccharomyces* genome database]) to identify homologs and infer functions for the mutated gene. The gene name was assigned following the SGD nomenclature.

### Phenotypic characterization.

All NAT^R^ transformants obtained with the binary vector containing the *ade2*::*NAT* gene replacement cassette were 10-fold serially diluted and spotted on mYNB supplemented with or without adenine (20 mg/liter). Melanin production was tested for the wild-type strain CBS 14141, two independent *lac2*Δ mutants, and a NAT^R^ strain obtained in the same transformation experiment on mMM with l-3,4-dihydroxyphenylalanine (l-DOPA) and on Niger seed agar as previously described (25, 27); *C. neoformans* wild-type strain H99 and the *lac1*Δ, *lac2*Δ, and double *lac1*Δ *lac2*Δ derived mutants (27) were used as controls.

Strains subjected to phenotypic characterization were serially diluted 10-fold in liquid mDixon and spotted (in a volume of 1.5 µl) onto the agar media. An iron-limited condition was achieved by the addition of bathophenanthrolinedisulfonic acid (BPS) at several concentrations (50, 100, 200, and 300 µM) to mYNB medium adjusted to pH 7 with KOH. Medium replete with iron was prepared by adding FeCl_3_ at a range of concentrations from 50 to 300 µM to the BPS-containing mYNB. In a similar manner, low-copper medium was prepared in mYNB (pH 7) containing the copper chelator bathocuproine disulfonate (BCS) at a range of concentrations from 50 to 300 µM, and high-copper medium was prepared by adding CuSO_4_ (50, 100, 200, and 300 µM) to the BCS-containing mYNB. These media were prepared according to Jung et al. (29) with slight modifications.

For stress sensitivity tests, mDixon agar was supplemented with the following chemicals: sodium chloride (NaCl [1.5 M]) for osmotic stress, hydrogen peroxide (H_2_O_2_ [5 and 10 mM]) for oxidative stress, cadmium sulfate (CdSO_4_ [10, 30, 50 µM]), and hydroxyurea (20 mM) for genotoxic stress, and sodium nitrite (NaNO_2_ [5, 50, and 100 mM]) for nitrosative stress.

### Phylogeny of laccase genes in the *Malassezia* genus.

The predicted amino acid sequences of the proteins Lac1 and Lac2 from *C. neoformans* strain H99 were used as query for tBLASTn searches against the genomes of strains of the 14 *Malassezia* species available in GenBank (9). The *Malassezia* sequences identified were retrieved from the genome assemblies, and the start and stop codons were predicted following BLASTx analysis against the annotated orthologs of *M. globosa*, *M. sympodialis*, and *M. pachydermatis* available in GenBank. These sequences were used as a query for BLASTx analysis of the H99 genome database (bidirectional BLAST), and *Malassezia* gene names were assigned based on the closest *C. neoformans* H99 laccase homologs. The full ORFs of the *LAC1* and/or *LAC2* genes were aligned used the software MUSCLE, and the phylogenetic tree was generated with MEGA 7 (http://www.megasoftware.net/) (47) using the maximum likelihood method (Tamura-Nei model, 5 discrete gamma categories) and 100 bootstrap replications.

## SUPPLEMENTAL MATERIAL

Figure S1  Maps of the binary vectors pAIM2 and pAIM6 used for *A. tumefaciens*-mediated transformation of *M. furfur* and *M. sympodialis*. RB and LB indicate the right and the left borders of the T-DNA that flank the *NAT* or *NEO Malassezia*-optimized cassettes (p*ACT1-NAT-*t*ACT1* and p*ACT1-NEO-*t*ACT1*); the multiple-cloning site (MCS) and the EcoRI sites used for subcloning procedures are also shown. *KAN* confers resistance to kanamycin for selection in *E. coli* and *A. tumefaciens*. Download Figure S1, TIF file, 0.1 MB

Figure S2  Representative example of biolistic transformation (A) and *A. tumefaciens*-mediated transformation (AMT [B]) experiments with *M. furfur* CBS 14141 carried out using the plasmids pAIM1 and pAIM2, respectively, both based on the dominant drug marker *NAT.* Selection was performed on mPDA supplemented with mPDA plus nourseothricin (100 µg/ml) for biolistic transformation and mPDA plus nourseothricin (100 µg/ml) plus cefotaxime (200 µg/ml) for AMT. Note that when biolistic transformation was performed, only clumps of *M. furfur* CBS 14141 were observed on selective medium (A); conversely, in the case of AMT, real NAT^R^ transformants (single colonies circled in red) were clearly distinguished from the background formed by the numerous clumps of *M. furfur* CBS 14141. Download Figure S2, TIF file, 1.3 MB

Figure S3  Phenotypic evaluation of the *lac2*Δ mutant derived from *M. furfur* CBS 14141. The wild-type strain CBS 14141 and three independent *lac2*Δ mutants were 10-fold serially diluted (starting from 10^−1^ to 10^−6^) and spotted in a volume of 1.5 µl onto the following media: mYNB, mYNB at pH 7 supplemented with bathophenanthrolinedisulfonic acid (BPS) at several concentrations (50, 100, 200, and 300 µM) with or without FeCl_3_ at a range of concentrations from 50 to 300 µM, mYNB at pH 7 supplemented with bathocuproine disulfonate acid (BCS) at several concentrations (50, 100, 200, and 300 µM) with or without CuSO_4_ at a range of concentrations from 50 to 300 µM, or mDixon agar alone or supplemented with hydroxyurea (20 mM), cadmium sulfate (CdSO_4_ [10, 30, and 50 µM]), sodium nitrite (NaNO_2_ [5, 50, and 100 mM]), and hydrogen peroxide (H_2_O_2_ [5 and 10 mM]). The plates were incubated for 4 days at 30°C and photographed. Download Figure S3, TIF file, 2.4 MB

Figure S4  Full ORFs of the *LAC1* and *LAC2* genes of the *C. neoformans* and *Malassezia* species used for the phylogenetic analysis. The sequences of the *LAC1* and *LAC2* genes of *C. neoformans*, *M. sympodialis*, *M. globosa*, and *M. pachydermatis* were downloaded from GenBank. The laccase-encoding genes *LAC1* and *LAC2* of the remaining *Malassezia* species were retrieved from the nonannotated available genomes. The start and the stop codons, which are highlighted in green and red, respectively, were predicted based on similarity searches through BLASTx analysis against the annotated orthologs of *M. sympodialis*, *M. globosa*, and *M. pachydermatis*. Download Figure S4, PDF file, 0.1 MB

Table S1 Sequences of oligonucleotide primers used in this study. Table S1, DOCX file, 0.01 MB
