# Supplementary figures and images for: Gene Function Analysis in the Ubiquitous Human Commensal and Pathogen Malassezia Genus
Source: mBio. 2016 Nov 29;7(6):e01853-16. doi: 10.1128/mBio.01853-16 (PMC5137500; doi:10.1128/mBio.01853-16)

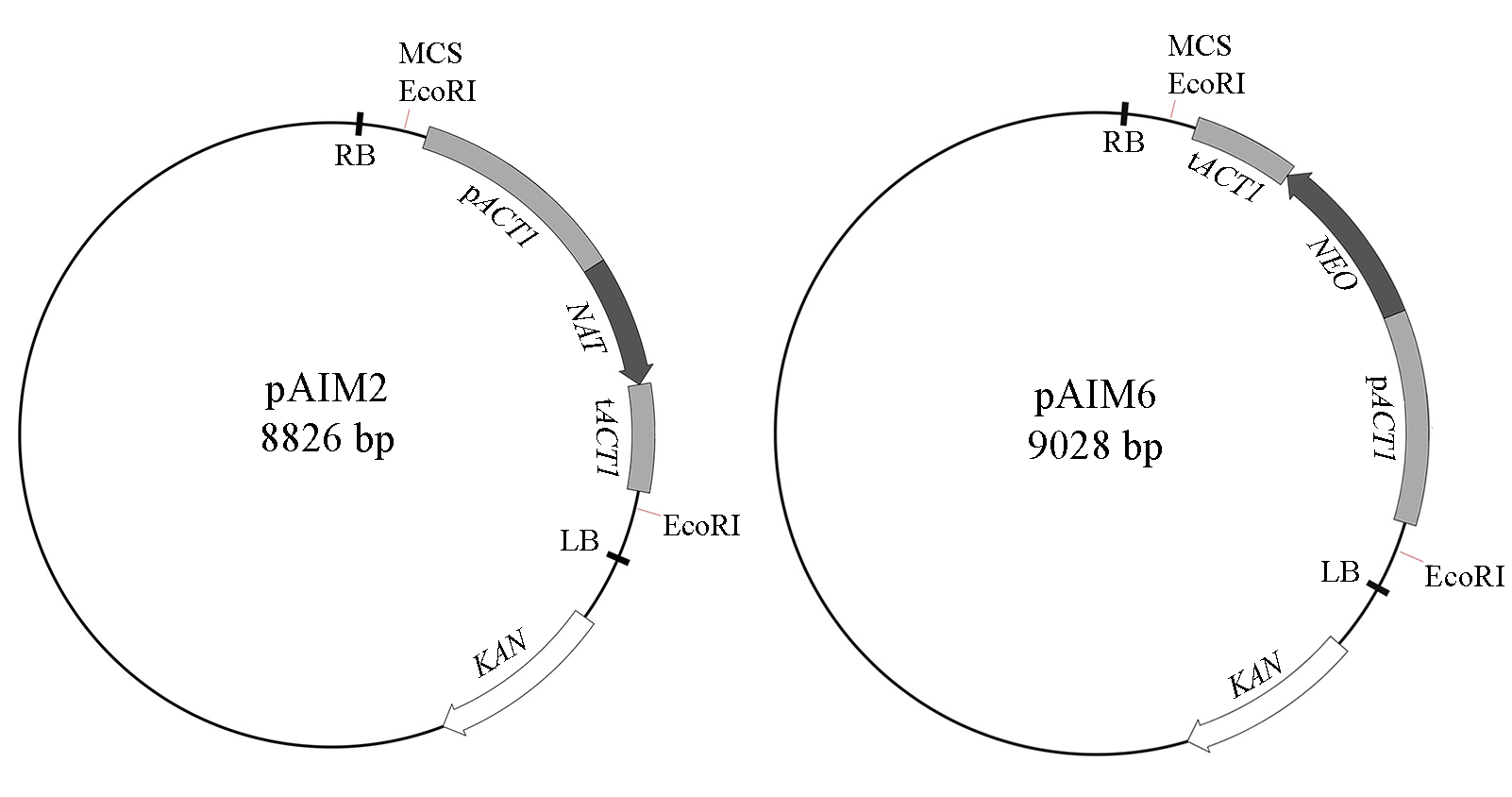

Supplement: Figure S1 — Maps of the binary vectors pAIM2 and pAIM6 used for A. tumefaciens-mediated transformation of M. furfur and M. sympodialis. RB and LB indicate the right and the left borders of the T-DNA that flank the NAT or NEO Malassezia-optimized cassettes (pACT1-NAT-tACT1 and pACT1-NEO-tACT1); the multiple-cloning site (MCS) and the EcoRI sites used for subcloning procedures are also shown. KAN confers resistance to kanamycin for selection in E. coli and A. tumefaciens. Download [file mbo006163076sf1.tif]

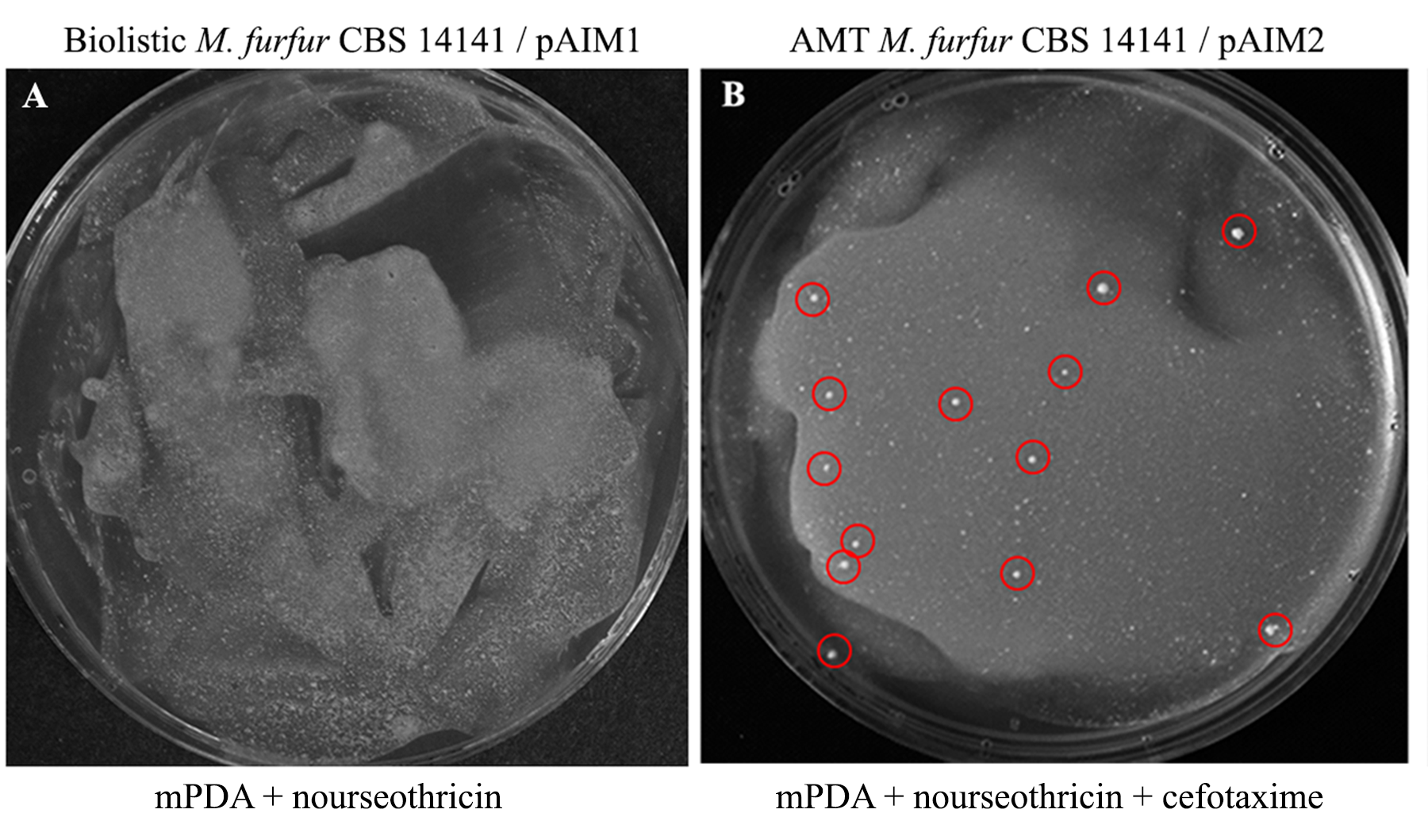

Supplement: Figure S2 — Representative example of biolistic transformation (A) and A. tumefaciens-mediated transformation (AMT [B]) experiments with M. furfur CBS 14141 carried out using the plasmids pAIM1 and pAIM2, respectively, both based on the dominant drug marker NAT. Selection was performed on mPDA supplemented with mPDA plus nourseothricin (100 µg/ml) for biolistic transformation and mPDA plus nourseothricin (100 µg/ml) plus cefotaxime (200 µg/ml) for AMT. Note that when biolistic transformation was performed, only clumps of M. furfur CBS 14141 were observed on selective medium (A); conversely, in the case of AMT, real NATR transformants (single colonies circled in red) were clearly distinguished from the background formed by the numerous clumps of M. furfur CBS 14141. Download [file mbo006163076sf2.tif]

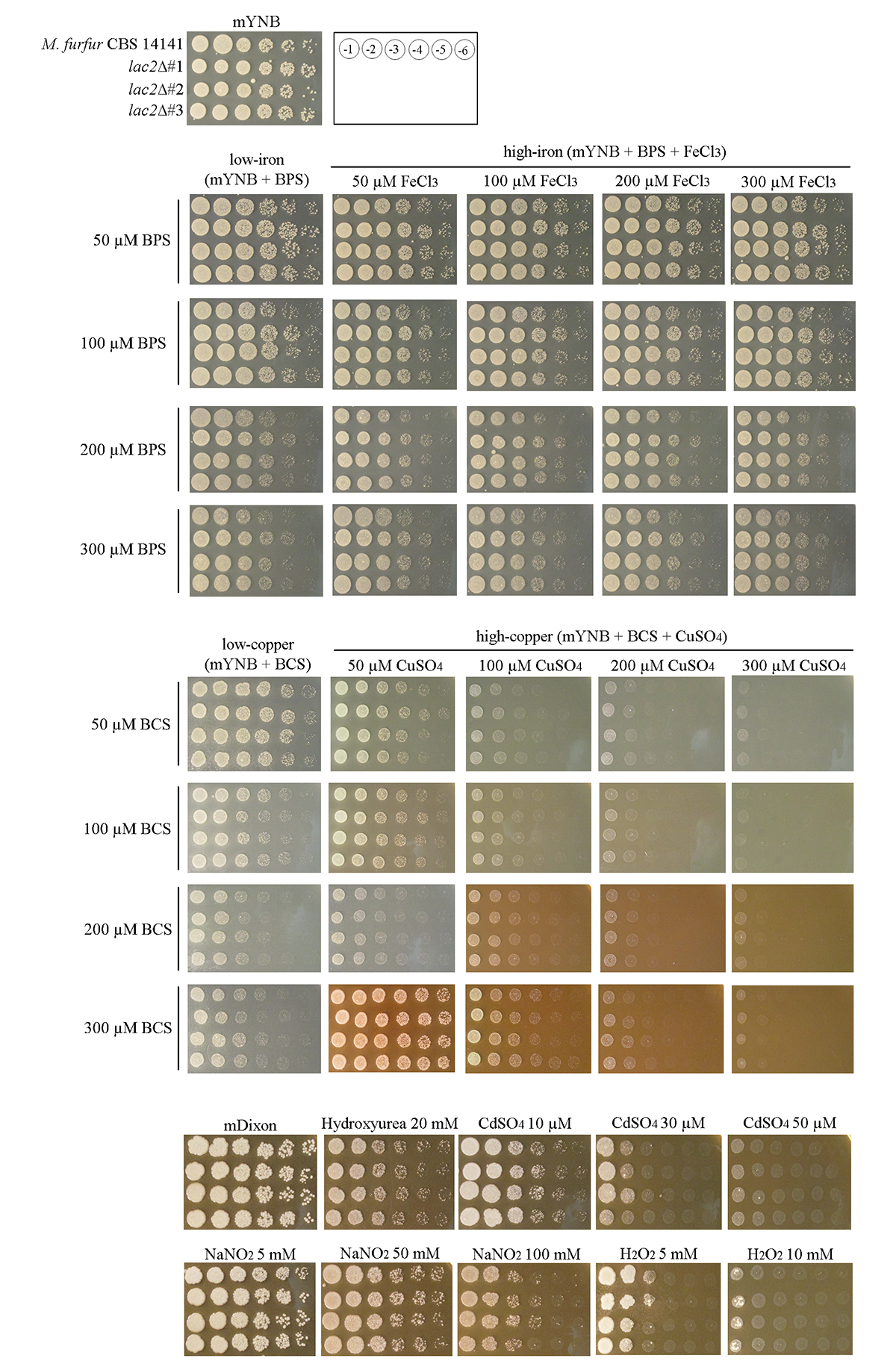

Supplement: Figure S3 — Phenotypic evaluation of the lac2Δ mutant derived from M. furfur CBS 14141. The wild-type strain CBS 14141 and three independent lac2Δ mutants were 10-fold serially diluted (starting from 10−1 to 10−6) and spotted in a volume of 1.5 µl onto the following media: mYNB, mYNB at pH 7 supplemented with bathophenanthrolinedisulfonic acid (BPS) at several concentrations (50, 100, 200, and 300 µM) with or without FeCl3 at a range of concentrations from 50 to 300 µM, mYNB at pH 7 supplemented with bathocuproine disulfonate acid (BCS) at several concentrations (50, 100, 200, and 300 µM) with or without CuSO4 at a range of concentrations from 50 to 300 µM, or mDixon agar alone or supplemented with hydroxyurea (20 mM), cadmium sulfate (CdSO4 [10, 30, and 50 µM]), sodium nitrite (NaNO2 [5, 50, and 100 mM]), and hydrogen peroxide (H2O2 [5 and 10 mM]). The plates were incubated for 4 days at 30°C and photographed. Download [file mbo006163076sf3.tif]
