# Supplementary material for: Gene Function Analysis in the Ubiquitous Human Commensal and Pathogen Malassezia Genus
Source: mBio. 2016 Nov 29;7(6):e01853-16. doi: 10.1128/mBio.01853-16 (PMC5137500; doi:10.1128/mBio.01853-16)
Supplement: Figure S4 — Full ORFs of the LAC1 and LAC2 genes of the C. neoformans and Malassezia species used for the phylogenetic analysis. The sequences of the LAC1 and LAC2 genes of C. neoformans, M. sympodialis, M. globosa, and M. pachydermatis were downloaded from GenBank. The laccase-encoding genes LAC1 and LAC2 of the remaining Malassezia species were retrieved from the nonannotated available genomes. The start and the stop codons, which are highlighted in green and red, respectively, were predicted based on similarity searches through BLASTx analysis against the annotated orthologs of M. sympodialis, M. globosa, and M. pachydermatis. Download [file mbo006163076sf4.pdf]

>*C. neoformans LAC1*

ATGCGGGGAGTAGTCAAGCTCTTCTTTCTATCTTGTTCCCTCGTTTCGCTGGTCAGCAGCGAGGAGACTGGCAAGT
GAGTTAATTTCGTTGAGCTTCACAAGATCCAGGGAAGGTTAATCATTGTTCAGGTCGCCAACCGCGAACTATGACC
ATTATATGCCGAAGGCGACAGCAACCATTGATCCTAGTGTATTCGCTCTTTCAAATGACTTTGAAATAACAGATGTT
CCGACGACGAGGGAGTATACCTTCGATATCACGTGAGTCAGAATCGAAAGGTTGCGTATTTTAATCTGACGCACA
ATTGCAGCAAAGCGTTGGCCAGCCCTGATGGTTATGAACGAGAGGTTTACGTTGTCAACAACATGTTCCCTGGACC
TGTGATAGAGGCTAACACCGGGGATACTATTATCGTACATGTCAACAATCATTTGGAGGAAGGACAAAGTATCCG
TAAGTATCAGCTGATATCCTGCTCATATCGAAAAGTACTTTTTAATGTACTAACTCTAATCCAGACTGGCATGGTTT
GCGGCAGCTTGGCACGGCTTTCATGGACGGTGTCCCTGGTATAACACAGGTAAGAATCCTTAGGAACCACAGGAA
TCGGTCAACTTATAAACTGCATAGTGTCCTATTCCCCCTGGAAGCTCATTTACCTACCAATTCACCGTAAGCCATCA
GTCAGGCACGTTTTGGTGGCATTCCCATTATTCCAATTCCATGGCCGACGGCATTTGGGGCCCGTCAGTTTTCCTGA
CTTTTGATAACAAGTTATCACAGCTGATAGGATACCATAGCTTAATTATCCATTCGCCCAATGAACCCCTCCAAAGG
GGACGAGACTATGACGAGGATCGAATCGTTTTTATAACTGACTGGGTGTAAGTATGACCGTTCTTTGGTGAGGCTC
AAGCTAAACATTGGTGATGGCAGGCATGACAACTCAGAAGTCGTTATTGCAGCTCTAGCTACTCCAGAAGGGTAC
AAAGGAGTGAGTAAGAGTTTGGAGATTAATAGTCCGATACTGACTGTAAGAAGAGCCCTGCTCCGCCACAAGGTG
ATGCGATTCTCATCAATGGACGTGTACGTGATTTTACTGGCAATGGCGATAACCCCCAAGTGGGCTTACTAACTCTT
ACCGCTAGGGCCAAACCAACTGCACAGCCACTGGTTCCTCCTCATGCACCTATCCGCCTCCTCCCGAGATTCACGTG
CCAGTCAATTGCAGGGTTCGTCTGCGCTTTATCAGTGCGACCGCCCATCCCATGTACCGCATAACTATCGACAACCA
CCCTTTGGAAGTTGTGGAAACCGACGGTACAGCCGTCTATGGGCCCACAGTCCATGAAATCTCCATTGCACCTGGG
GAACGGTACTCTGCAATTATCAACACCTCAGAAGGGAAGGAAGGTGATGCGTTCTGGCTGAGGACAAGTGTTGCT
CTGGGCTGTATGTTTGGTGGAATAGATCAGGTGGGATTGGCGGTTGTGAGGTATACGGGTAATGGAATGGTTAG
TACTGAAGAGCCTCAAACTACTGCTTGGTGAGTTACTGGGAAACCTGTAGGTTGAACTGCCGCTGACAATATATTC
AGGAGTGATCTAGCGGGAGCTACAACTCCTTGTGCTGGACTGGACCAAACATATACTCTTTCGTGAGCACTGGTCG
ATTTCTTTTGTTTGATTCTTACTAATAAACCACGCAGACCACGAGAGAGTTTTAGTGCACCTCGTGAATTTTCACAA
AGCCATGTCTTCAATAGCCAGCGAGGAGCCTTTGTGAATGTTTATGGCAACACCTTCCAAGGTTATGTATGTAATTT
TCCATTTTCCAGTATTACATTTAAGAAAAAAAAGGTGCTGACAGTCATCTAGGGGTTTAACAATATCTCATATCAGA
ACCAAATCTTCAACCCTCTACTTTCAATCGTCCAACGCGGTGGCTCTTGCGAGAGCACACTAGTAGCCAGTACAACT
TTCCCCGACCTCGGATCAGGGAACATTATCATCAACAATCTTGATGGCGTTATCGACCATCCTTACCACCTGCACGG
CAACGAGTTCCAGGTGATAGGACGAGGAACTGGAGCTCTCAGCCTTGATAACCTGACAAATATTGACTTCAATTTG
GACAACCCTGTGAGAAAGGATACCCTCTGGATACAGGGCGGAAGTTGGGTGGTACTGAGGATCACGACGGATAA
CCCTGGAGTTTGGGCCTTGCACTGTCATATTGGGTGGCATCTTACTGAGGGAAAGGTAAGTTTTTACCCTTGGCTC
CGACATTAACCCATTAAACTTATTTATATAGTTGGCTGTGGTTGTCATTCAACCAGGTGCGATTGGACATATGGAG
GGCCCCGAGTCTTGGACGAATGTGAACCCAGCTTCTTGATTTCAAAGAACTTTTGCTAACTTATTTCGCCCTTTAGC
TCTGTGCTAACACTGATCCCAATGCATTTGGACCCGCACGACGCTCACCTTCTCCATCTATTCAATCCTCTAAGACAT
CCACTTTCCAGTATCTGCGCGAAGTGAAAGGGAAGGTCGTTAAACGTAGAGGTGCTCGAGAGGCGTGA

> *C. neoformans LAC2*

ATGGGAGGCATAATCAAGCTTTCCTTCCTCTTCTGTTCCCTCATTTCTTTGGTCAACAGCGAGAATACTGGCAAGTA
AGTTACGTCGTCGGGTCGCGGAGCTCCCGAAAGGCTAATCACTGTCAGGCTGCCAACCGCGATTTCTGATCATTCT
GTGCCGAAAGCGACAGCAACCACTGACCCTAGTGTATTCGTTCTTTCAAATGACTTCGAAATAACAGACGTCCCGA
CGACGAGAGAGTATACCTTCAACCTTACGTGAGTCAATCCTTAGGGGCCTGTTCGGCAACCGCAAAAGTGGTGAC
TTTGCCAAGGTCACCAGCCAAATTTGATTCTGTTCAAAAAAGTCAGAACGTGAGATCACCGCCATGACTTTGGCCC
TTTCGCCCTGTTCGGCAACGAATATGACCCCTCTGAAAATCACAGAAATTTTATGAAACGTTCGAAAAGATGCCGA
ACAGGTTCTATTCTTAAACCTCAAGATCGGCTTATCTAAAGCTAAAACAATTGCAGTGAGGCTTTGGCCAGCCCTG
ACGGTTATGAACGGCTTGTTTATGCCGTCAACAACATGCTCCCTGGACCTGTGATAGAGGCTAACACCGGGGATAC
TGTTATCGTACATGTCAACAATTATCTGCATGAAGGACAGGGCATCCGTAAGTATATCTTCTCATGAAAAGGTGAT
GTCATGCCTCCATTGTCACTGACTTCCAATTCAGATTGGCATGGTTTGCGACAGAATGGCACGGCTCTCATGGACG
GTGTACCTGGTATAACCCAGGCAAGTATTCTTGGAGAGGACAGAGAGTCCATATGACTGAGATGTCTATTAGTGTT
CTATTCCGCCTGGAGGCTCATTTACCTACCAATTCACCGTAAGCCATCAATCAGGCACGTTTTGGTGGCATTCCCAT
TATTCCAATTCCATGGCCGACGGCATTTGGGGCCCGTCAGTTTTCCTGACTTTTGATAACAAGTTATCACAGCTGAT
AGGATACCATAGCCTAATTGTCCACTCGCCCAATGAACCCCTCCAAAGGGGACGAGACTATGACGAGGATCGAAT
CGTAGCTGTAACTGACTGGATGTAAGTATGGCTATATTGTTTGATGAAACTCAGCTGAATACTGGTGATGATAGGC
ATGATGAATCAGAAACCATCGTTGAAGCTCTAATCAGCTCAGAAGGGTATAGAGGAGTGAGTAAGAAGAATTCG
GAGATTAATAGTCCGATACTGACTGTAAGAAGAGGCCTTTTCCGCCACAAGGTGATGCGATTCTTATCAACGGACG
TGTACGTGATTTTACTGGCAACGACGTAACCCCCAAGTGGGCTTACTAACTCTTACCGCTAGGGCCAAACCAACTG
CACAGCCACTGGTTCCCCCTCATGCACCTATCCTCCTCCTCCCGAGATTCACGTGCCAGTCAATTGCAGGGTTCGTC
TACGCTTTATCAGCGCTGCCTCTCACCCCATGTACCGCATCTCTATCGACAACCATTCCATGGAAATCGTTGAGACG
GATGGCACTGCTGTTTACGGACCTACCATTCATGAAATTTCTATCTCGTCTGGAGAACGGTACTCTGTAATTATCAA
TACCACCGAAGGAAAAGAAGGGGATGCGTTCTGGCTGAGGACAAGTGTTGCTCTGGACTGTATGGCGCAAGGGG
TTACTCAAGTGGGGTTGGCGGTGGTGAGATATACGGGTAATGGCTCTATTACTACCGCTGAGCCTCGAACTGAAG
CTTGGCGAGTCATTAAGAAGCCTACGGTGGAAATTCTTGCTGACAACATATTTAGGACTGATTTAGCGAGACCTGA
CACTCCGTGTGTTGGGCTTGATGAAATGTATCATCTTTCGTGAGGGCCTTTCCTTCTCCTTGCTTTGCCAAGTTCACC
TCTAACGGTGCTCAGGCCACGAGAGCTTGTGAATGCGTCCCAAACAGCTTTGGAGAGTCGTGTTCTCGATAGCAA
GCTGGGTAAATTCGTGGACGTCTATGGTAACTCCTTCGAAGGTTACGTATGTGATTTCCAGTACTATTCTAAAGGA
AGAAGACGCCAACAGTCGCTTAGGGATTCAATAATGTCACATACCAGAACCAAATAAACGACCCCTTACTTTACGT
CGTCCAGCGTGGTGGCACTTGCAACAGCTCATTAATAGCCAACGCAACTTTTGCAGATATCGGTCCTGTTAACATC
ATCATCAACAACCTAGATTCCCATATTGGTCACCCTTACCACATGCACGGCACCGAGTTTCAGTTGATGGGACGAG
GTACTGGAGCTCTCACTCTTGATGACCTGCCAAATACTAATCTTACTTTGGACAATCCTACGAGGAAGGATACGATT
TGGATGCAGGGTGGAAGCTGGGCATTATTGAGGATCATTTCTGATAATCCCGGGGTGTGGGCCTTGCATTGTCAT
ATCGGATGGCACCTAGCCAAAGGGAAGGTGGGATACAGTGTTACTCCTCCAACTGGTTTATTCACTAATTGTTTTG
TGCAGATGGCTGTGGTCGTTGTCCAGCCAGAAGCGATCAAAAAGATTCAATGGCCTGAATCTTGGATGGATGTGC
GTCAAATTTCTTCATAAGCAAATTACTGACTGACTCATTACTTTACTCAGCTTTGTGCTAACACTGATCCCAACGCAT
TTGGTCCAGCGCGGCGTTCAGTTTCTTGA

>*M. sympodialis LAC1*

ATGGACCCTAATTATAATCACTCAGCTCTGCCACAAGAACGGCATTTTGACTGGACTGTTGAGAGAAAGGTGGTG
GCCCCAGACGGCGTTCCTCGTTTAATGTACACTATCAATGGCCAGTTCCCTGGACCTATCGTGGAAGTGACAGAAG
GTGACACCGTTGTCGTGCGAGTTCGAAATCATATCTTCGATGACTATCAAGTGCCCGAGCCTCCCATTACATCTCGC
CTCCATGATGTGTTTCCAGAGGGCACAGACCGCAAATTTTCCCTTCATTGGCATGGTCTTTCTATGCGTGGACAGCA
AGTAATGGATGGCGCAGCCTCTTTTACATCCTGCCCATTAAAACCAGGAAACGAGACGGTGTATCGATTCACTGTG
TTTCACGAAGATGTGGGTACACATTGGTACCATTCTCATGTCGGCACGTCGCGTGCGGATGGTTTGTGGGGAATGT
TAATTGTCCATGCTCGGGAAGATGAGCGGCCTTTATTCCAAGAGCATGCACTAGGTGCGAAAGTACACTGGGACG
ACGAAATTGCTGTGGCGCTTGGAGATCATTTCCATGTTATGAGTCCCGAAAGTCTGGCCGAATATGTTTCTATCCTT
TTGTCTAATGCAGAGCCCGTGCCTGACAGTGGTCTCATCAACGGAAAGCATGTATTCAGCTGCTACATGAGTCGGA
AAGAGGACGTTCCATGCCCCGCGGGTGATGTCGATGAGGTAGGCGAGTACGAAGTCTTCCGTCTCAACCCACAGC
GCTCCTATCGCTTGCGCCTGGTGAACGTTGGATCGGTGGCTGATGTTGTATTCAGTGTGGATGGTCACACGATGAC
CGTCATCGAGGCCGACGGAACGTTAGTGGAGCCCATGTCTGTGCACCGCATTCCCATTGCTCCGGGCCAACGATA
CTCCGTCATCTTGCACCGCGAACCGTTCAGCAAAGTTGATCGAGTTTGGATGCGAGCAGAAATGACTGCAGAATG
CTTCAAGTATCTAAATCCCGTGATGGATCCTATGGTCAAGGCGATCGTGGCTTACGATAAAAGCGTCACAGACGAT
GGCCATTGGTTATCGCCTTTGAGGATGCGGAGCTACCGTCGCACTGTGTCCGAACGCATTTTGTCACGACGTTGGC
GCTGGACGTTGCGGCCCACCAGTCAGGCATGGAGCCCCAACGTAACCGATGTGGGAATCCCTGAGGAGCCTTGCC
ATGACTTGGAGCCAGGGACACTTGTTCCATTAGTCGCTGATCCTGCACCCAAACTTGATGTCGGCCGTGGAGACCG
TCAGGTTTATGTCAATATTGAGGTGTTGAACCGCCACAAGTATTCGAAAGCACCGATGTCGTTCCTAAATATGTCTA
CGTGGCGCCCATACGGCCTCCAGGGGCCATCTAAACAGCCATTACTACACCGTATCTCGTACTCCAATCTGACGTC
GCCGGAAGAATGGGAACAGACCGGGCTCGTGAACCGCGAACATGAGCTAGTGGTAAGCACGCACCCAAGCAAGC
CCGTTGTTTTTGAATTGGTGCTATTTAATTACGATGATGGACCTCATCCTTTCCATCTCCATGGGCATAAGTTTTGGG
TGCTGCACACCGGCGAAATGGAGCGCTTGCGATTCCAGTATACCAAGGAGGTGGAGGACTCATTTGATCTGGCAC
GCGCCATGAAGCGTGACACGGTGGTGGTGCCTATGTTGGGTCATGCGGTGATTCGATGGGTAGCCGACAATCCTG
GTGTATGGGCCTTCCACTGTCACATGAATGTGCACCTTGCCAGTGGAATGGCTATGGCCTTTGTTGAGCAGCCCCA
GGTCCTCCAGGCTCGCCCCCCTGTGCCCGCCACATGTCAATAG

>*M. sympodialis LAC2*

ATGGACCCTAGCTACAATTCTACGGAGCTACCTCGTGAGCGTGTTTTCGACTGGACAGTGGAGCGCGCAGAGCAG
TCGCCTGACGGTATCCCGCGATTAATGTACACCATCAATGGCCAATTCCCAGGACCCATCATTGAGGCTACAGAAG
GTGATACAGTGGTGGTTCGCGTAAGAAACCACATTTATGATAACTTTACCATACCGCCGCCGCCTCTCTCATCCCAA
CGAGATGATGTGCATCCGGACGGGACAGACCGTAAGTTTACGTTCCACTGGCATGGCCTCTCCATGAGGAACACA
CAAGTCATGGATGGCGCATCAGCGTTCACTTCCTGTGCTTTACTTCCAGGGGAAGAGCGAGAGTACCGATTCCTCG
TGCATCCGGAAGATGTTGGAACGCATTGGTATCATTCGCATGTCGGCACGTCACGAGCCGACGGCTTGTGGGGTA
TGTTCATTGTCCATTCGCGTACCGATGAGCGTAAGAAACTTTCCACGCGCTTTCCAGATGCAAATACCCATTGGAAT
GAGGAAGTGGCCATTGCCTTGGGTGATCACTACCATCAATTTGGTCCCATTAGCCTTGGCTTTTACGTCTCGCGTTG
GCTTCAGAAAGCTGAGCCGGTACCTGAAAATGCACTGATAAATGGCAAGCATGTATTTTCTTGCCACCACAGCCGT
CTAACTGGTGTACCCTGCCCCGCTGGTGACGTCGATCAAGTGGGTGAATATTCTACCTTCAGTCTGGATCCCAAGC
AGGCCTACCGTCTTCGACTCGTTAATGTGGGTGCACTCGCCGACATCACTTTTAGCGTCGATGGTCATACCATGAC
GGTCATTGAGGCGGATGGTACATTGGTCGAGCCTGTGCGTGTCCATCGCATTCCAATTGCTCCTGGTCAACGCTAC
TCGGTTATTCTGCACCGGGAGCCCAAAAGCAAAGACTCGCGAGTATGGATGCGGGCTGACATGTCCGCAGAATGT
TTCCAGTACACGAACCCTGTGATGGAGCTCGGCGCCAAGGCTATCATTGCATACGATACAAGGCAGAGTGGAGAG
GAGAGCAAGGATGATTGGAAATCCTTGCTGCGCCTACGAAGCTATAAGCGTGGCTTGACAGACAGGATCTGGCAC
GGCAGCTGGTTCCAGAGCCAATTACCTGCGACATTGCCGTGGAACCCCAATGTTACTGACACAATTCTTCCGGAAG
AACCGTGCCATGACTTGGAGCCGGACACGCTAGTTCCTCTTATCCCCGATCCTGCACCCCCGCTCCGGCTTGACCA
GGGTGACCACCGTGAGTTCGTCTTTGTCACAGCCCCAATCCTAGAGCGATACGGCGTTGTACCTATGGGTTTCATG
AATGGCTCAACGTGGCGCCCATATGGACGCCATGGCCGTGAACGCCAGCCCCTGTTGCACCGCATCTCTTATGCTA
ACACCTCCACTGTGCAAGGCTGGCATGATCAAGATATCCTTGATCCCAGTCATGAGCTCGTGGCGAGTCCGCATCC
CAGCCGACCAGTAGTTATGGAAATGGTCATCAACAACCAGGACGATTCCCCGCACCCATTCCACCTGCATGGACAC
AAGTTTTGGGTGATGGAGACAGGGGAGGCAGACCCCAATTTCGGAGGGTTTGACTACTATGAGGATGTGGGCCA
AGTCTACAATTTGAACCGGAAGATGAAACGAGACACGGTGATTGTGCCCATGATGGGACACGCAGTCATCCGATG
GGTGGCTGATAACCCAGGCGTGTGGGCCTTCCACTGTCACATGCTTGTCCATTTGGCGAGCGGCATGGCCATGGC
GATTGTTGAGCAGCCAGAGGTTCTGCAAGCTCAGCCCCCAGTGCAGCGGACTTGTTCGTAG

>*M. globosa LAC1*

ATGGACGTAACCCTCCAACAGACGCTACAACATGCATGGGACGAGAGTTGGAGCGCGTTGCGCGTGTACACTGCG
CTCAAGCAGCAATCTGATCCCATGGCGCTGGATGCGTCATTTAATCCAAATGAAGCTCCTCGTGAGCGTGTCTACG
ACTGGACTGTGGACCGTCGGATCCAGGCACCTGATGGCGTGCCGCGTCTCATGTACACAATCAATGGAAAATTCC
CAGGACCTACCATACAAGCCACAGTGGGTGACACAGTTGTTGTCCACGTTCGTAATCATATTTGGGATGACTATCA
AGTTCCAGAGCCTCCTATTACTTCTAAGTTGGACCACGTTCATCCCGAAGGCACTGATCGCAAGTTTGCTATCCACT
GGCATGGCCTGTCAATGCGAGGAACCCAAGTAATGGATGGTGCCGCCGCATTCACATCCTGTCCGTTAAAACCAG
GCAACGAAACAACATATCGTTTTGTTGTGCACCCGGAGGATGTCGGCACGCATTGGTACCATTCACATGTGGGTAC
GTCACGCGCCGATGGTTTGTGGGGTATGTTGATTGTGCATGCCCGGGAGGACGAGCGTAAAGTCCTGAAGGAAC
GTGCACCAGCGCATGAAACACACTGGGATGAGGAAGTCGCCATTGCTGTGGGCGATCACTTTCATGAAATGAGCC
CCCAGAGTCTTGGGAAGTACGTCTCCATTATCTTGTCCAGCGCTGAGCCTGTGCCCGACAGCGGGCTCATTAATGG
TAAACACATTTTCTCCTGTAACATGTCGCGCATAACCGATGTGCCTTGTCCCGCAGGTGATAAGGATGAAGTGGGA
GAATATGCACAGTTCCATTTGCGTCATGACCAGCAATATCGCCTGCGTCTTGTGAATGTAGGCTCACTAGCTGACG
TCATGTTTAGCGTCGACGAGCACACTATGACAGTCATTGAGGCGGACGGTGTGCTTGTCGAGCCTATGAAAGTAC
ATCGTATCCCCATTTCACCGGGGCAGAGATACTCAGTGATTCTGCACCGTGAGCCGAGCAGCAAACGGCACGAGC
GCGTTTGGATGCGCGCAGAACTTGAGGGTGAATGCTTCAAGTATATGAATCCCGTTCTAGATCCATTCCTCAAGGC
CATCGTCACTTACGACGTTAACATGCCGCCACTATCGGGCGGCTGGCTGTCGCCACTCCGCGCTCGGCAGCTCTTT
GGCCAACAGGCGACAGAAAAGCATCGTCGGTATACGTCGGCATCAAAGCGGCCTACCACCCAAGCTTGGAGTCCA
GATATTAAGGACGAAGGGATCCCATCCGAGCCATGCCACGACCTGGAGCCTGGTACACTTGTGCCGCTTATTCCCG
ATCCAGCGCCAGAGTTTATTCCTGAGCGAGGTGATCGACGCGAGTTCATCAACATTGAGGTATTGACGCGTCAAA
AACTTCGCGTGCCTATGGCCTATATGAACTTGACGTCTTGGCGTCCTTACGGCGCGCGTGGACCCTCGCAACAACC
ACTTCTACATCGTATCTCGCATACAAATTACTCGACGGTTGAGGACTGGGAGCAGCATGGCCTAGTCAACCGTGAG
CACGAATTGGTCGTGAGTCCTCACCCTACCAATCCCGTCGTGTATGAGCTCGTTCTCATCAACTTGGATGATGGTCC
GCATCCTTTCCATTTGCATGGCCACAAGTTCTGGGTTTTACATACAGGTGAGATGGAAATTCCCGCATATCGCTATA
AGCCTGAAGTGCAGCGAGAGTTCGATTTGCAACGTGCTATGAAGCGTGACACAGTCGTCGTTCCTATGCTGGGAC
ATGCAATAATTCGTTGGGTCGCTGATAACCCAGGTGTGTGGGCTTTTCATTGCCATATGCTTGTTCATCTCGCGAGC
GGCATGGCAATGGCTATCGTTGA

>*M. globosa LAC2*

ATGGGATCCCAAAGCAGAGCTCGTACCGTGGGATACGGTCTTGTGTTCTTTCTGGGCTGTGCTTTGACTAGCGCAG
TTCTGTGGCTTTTGTACGCAGATGTTGACCTGCATGGATCCGTTCACAGTGCGTGGGATGCTAGTACGGGTGCCTT
TCGTCTGGGACGAAACACGCAGCTGGTTCCTGCCACCGAAGTGACAACAGGTCCATTAGCCATGGACCCCTCATAC
AATGCAAGTGCTCCTCCTCAAGAAAGGTTCTTTGATTGGACGGTAGAGCGTGCTGACATGGCACCCGATGGTGTG
AAGCGCCTCATGTATACCATCAATGGGCAATTCCCGGGGCCCATGATGGAAGTGACCGAAGGCGACACTCTTATT
GTGAAGGTCCGTAACCACATTTTCGACAACTACAAAGTACCGCCTCCTCCAATGTCGTCCAAACTCCTCGACGTTCA
CCCAGAGGGTACTGACAGAAAAATATCTATCCACTGGCACGGCCTGTCCATGCGGGGTATGCAGGTCATGGACGG
AACATCTGCCGTAACATCATGTGCACTTACACCAGGTGACGAGCATACATACCGCTTTGTCGTGCAGCCAGAAGAC
GTCGGCACGCATTGGTACCATTCACATGTGGGTACGTCACGCGCCGATGGTTTGTGGGGCATGTTGATTGTGCAT
GCCCGGGAGGACGAGCGTAAAGTCCTGAAGGAACGTGCACCAGCGCATGAAACACACTGGGATGAGGAAGTCG
CCATTGCTGTGGGCGATCACTTTCATGATATGGGCCCTGAGAGCTTGGCCCGTTACGTTTCGCGCTGGCTCCAAAA
AGCTGAACCGGTTCCGGAAAATGCGCTGATCAATGGAAAGCACATTTTCAGCTGCCACCACAGCCGGCTCTCTGGT
GTTCCGTGTCCAGCTGGTGATGTGGATCAGGTGGGCGAGTACACTTCATTCCACTTCCGGCCTGATCGTAACTACC
GTCTGCGGCTTGTGAATGTCGGCTCGCTGGCTGATATCACATTCAGCGTCGATGGCCACACGATGACGGTTATTGA
AGCCGACGGCACTCTAGTTGAGCCAATGAAGGTGCACCGCATCCCTATTTCGCCCGGTCAGCGCTACTCGGTGATC
CTACACAGAGAGCCTGACGCCAAGATGGATCGGATGTGGATGCGAGCTGAAATGTCGCATGAATGTTTCCAGTAT
ACGAACCCGGTCATGGAGCTGGAAAGTAAGGCAGTCGTTGCGTATGATGGAAAGACAGCATCTTGGAAAGACTC
GCAGAATCATATGTTCCCGCTTCGATTGCGGAGCTACCGGCGGAAAGTGTCTGAATTCATTTTCCAACATAAGCGA
ACATTGAAATTACCGACGTCCAAGGCATGGAGCCCAAATGTCACGGATGTTGGTATTCCAACAGAGCCTTGCCATG
ATCTTGAGCCCGATACGTTAGTACCACTCATTCCTGACCCGGCGCCTGAACTCCATCTTGACCAGGGCGATAAGCG
AGAGTTCGTCTACGTGACAGTGCCGATCCTAGAGAAATACAGCATTGTACCTATGGGATTCATGAACGGATCTACC
TGGCGACCGTACGGTCATCGTGGCCGCGATCGCCAGCCCATTCTGCACAGGATTTCACATGCAAACAGCACTGAA
GTCGAGGACTGGTACAACTATGATGTGCTTGAACCCTTACATGAACTTGTCGCTAGTCCGCATCCTAGCAAGCCGG
TCGTGTTTGAGCTGGTGATTAACAACCAGGACGATTCACCTCATCCTTTCCATCTTCACGGACACAAGTTTTGGGTT
ATGGAAACGGGTGAAATGGATCCTCAATTTGGCGGTTTCGACTACTACGAGGATGTTGGTCAAGTCTACCCATTG
GATCGTCGCATGAAACGCGACACCGTAGTCGTTCCAATGATGGGACACGCCGTTATTCGTTGGGTTGCCGACAAC
CCGGGCGTGTGGGCATTCCATTGTCACATGATGGTCCACCTGGCCTCTGGCATGGCCATGGCTATTGTGGAGCAAC
CTGCTCTGCTTCAACAAAATCCGCCAGTGCCAAGAGTTTGCAAATTA

>M. pachydermatis LAC1

ATGGCGCTGAAGCCTAAGGTCGTGAGACGGCTCTTGCTACTCCTAGTCAGTACAGGAGTTCTGGGTCTTTTTGCTT
GGGTTGCTCTTACAGATCCGGAGCTCTCGCGGGCTACACGGCAGCTGTGGGACGACAATGTATGGAGCCATGCCT
TGAAAAGCACCAACACCCCTTTTGCGTCTTCCAAGAGTCCGATCGACATGGATCCCTCATATGTAAAAAGCGCAAG
TCCCGTGGAGCGCCGTTTTGACTGGACGGTACAGCGTGAGATCAAAGCACCTGATGGTATTCCGAGATTGATGTA
CACCATTAATGGCCAATTTCCAGGCCCCACTATCGAAGTAACGGAAGGAGATACTGTGGTCGTCCATGTGGTAAAC
CATATCTTTGACGATTATCAAGTGCCTGAATTCTTGTTCGATAATAAACTGGAACACGTCCATCCAACAGGTACCGA
CAAGCGTTTCTCTATACATTGGCATGGCCTATCAATGCGTGGCATGCAGGTCATGGATGGCGCTGCGTCCTTTACA
TCTTGCCCTATTAGACCTGGAAATGAGACTACTTACAAGTTTGTCGTGCAGAAGGAGGATGTGGGTACGCATTGGT
ACCACTCGCATGTCGGTACGTCGCGTGCAGATGGATTGTGGGGTATGTTCATCGTGCATGCCCGCGACGATGAGC
GTCAAGTGCTAGCTGAGCGTGCACCTTCGTTCCGTACTCACTGGGACGAAGAGATTACCATTGCACTAGGCGATCA
CTTCCACAAAATGAGTCCAGAAAGTCTGGGTCGATACGTCTCGCGCACACTCTCCAATGCGGAGCCTGTGCCTGAG
AATGGTCTGATCAATGGCAAGAGCATCTTTTCGTGTGAGATGAGTAAATTGACTGGCGTTCCATGCCCAGCAGGG
GATGACAAGGATGAAGTCGGTGAATATACAGAATTCCGTCTGGACCCTACGAAAACATATCGCCTGCGTCTTGTG
AATGTTGGCTCGCTAGCGGATATGACTTTCAGCGTGGATGGTCACACTCTGACAGTTGTAGAGGCCGATGGAACG
CTAGTCGAGCCTATGGAGGTACATCGTATACCCATTGCACCTGGTCAGCGTTACTCTGTGATCTTACATCGAGAGC
CTTCAAACCGCGATAAGCGTGTGTGGATGCGATCAGATATGTCGGGCGAATGTTTCAAGTACATGAACCCAGTCCT
ATACCCCTTTATTCGTGCCATTGTGACCTATGATGAGCCTGACACCTGGACAGGTGTAGGTGGCTGGCTTTCGCCA
CTACGTCATCGCAGTAATCGCCGTGCCATGTCAGAGCGTGTGTATGAGCAACGAACCCGAGGCCTGTCACGCATG
CTGCCATCAACGCGCCCATGGCTGCCTAATGTTACGGATGCAGGTATACCTACTGAGCCATGTCATGATCTGGAGC
CAGGTACGCTGGTACCGTTGATTCCAGACCCAGCCCCGGCATTGGACTTTGCCAATGGCGACAGGCGCGAATACG
TCACAATTGAAGTGCTAAACCGTCAAAAGTATCAAGTTCCCATGGCGTTTATGAACTTGACAACATGGCGTCCCTA
TGGTCGCCGTGGTGCTTCGCAGCAGCCGCTTTTGCACAGAATTTCGCACAGCAATGCTACAACACCGCAAGCATGG
ATCGAGCAAGGCTTGATCAACAAGGAGCATGAGCTTTTGGCTGTAACGCATCCGTCCAAGCCTGTCGTATTTGAAC
TCGTTTTGAGGAATTTGGACGACGGCCCGCACCCTTTCCACCTGCATGGCCACAAGTTCTGGGTCCTTCACACAGG
AGAGATGGAACGTGTGCGTTTCCGGTACACGAAGGAGCTGGAAGACTCTCTAGATCTATCGCGCGCCATGAAACG
CGACACAGTCGTCGTGCCCATGATGGGCCACGTGATTATTCGCTGGGTCGCAGACAATCCTGGTGTATGGGCCTTC
CATTGTCACATGTTGGTCCACCTCGCGAGTGGAATGGCCATGGCGTTTGTGGAGCAGCCGAGTGTCCTCCAAGCG
CATCCGCCTGTGCCCGCCGCCTGTCAGTAG

> *M. pachydermatis LAC2*

ATGGACCCAAGCTATAACACGAACCAACCGCCTCAGGTCCGTATGTTTGACTGGACAGTGGAACGCAAAGACTTG
GCGCCGGATGGTGTGACGCGCCTCATGTACACGATTAATGGCGAGTTTCCAGGCCCCACGATCGAGGCCACGGAA
GGCGATACGGTTGTGGTACGTGTACGCAATCATATCTACGATGAATATACGGTGCCACCCGCCCGCATGTCCTCCA
AGCTGGAGGATGTGCATCCCGAAGGAACAGACCGTAAGTTCTCTTTCCATTGGCATGGGCTATCGATGCGTGGCC
AACAAGTGATGGACGGTGCCTCCGCCTTCACGTCGTGTGCACTGAAACCAGGTGACGAGAAAGAATACCGATTTC
AAGTGACGAAGGAAGATGTGGGTACACATTGGTACCACTCGCATCTTGGTACGTCACGCGCGGATGGATTGTGG
GGCATGCTCATTGTGCATGCCCGTAGTGATGAGCAAAGCACTATAAAAACGTATGTCCCTGCATCTTCAGTGACAT
GGGATGAGGATGTGGCGATTGCCTTGGGTGATCACTTTCACGATATGAGCCCTGTGAGCCTCGGTATGTACGTCTC
GCGCTGGCTCCAAAAGGCAGAACCTGTCCCTGAGAATGCCCTGATCAATGGCAAGAATGTATTTTCGTGTATGCAC
GCGAACTTATCCGGCGTACCTTGCCCTGCTGGTGACAAAGACCAAGTCGGCTCGTACTCCCACTTCCATTTCGATGC
GGACAAGACGTACCGTCTTCGCTTGGTCAATGTCGGCTCCCTGGCCGACATTACTTTTAGTGTGGACGAACATGTG
ATGACAGTCATTGAGGCGGACGGTACACTAGTAGAACCGATCCAGGTCCACCGCATTCCCATTGCTCCAGGCCAG
CGTTACTCGGTAATCCTTCATCGGGCTTCAAAACGACCCTCTAAGAATGTATGGATGCGCGCCGTCATGTCAGCCG
ACTGTTTCCAGTACACCAACCCTGTGATGGAATTTGAAGGCAAAGCTATCGTGTCTTACGACCGTATACCTACGAA
ATGGAAAGGCGCTGGTGGATGGCTGGCACCGCTGCGACAGCGAAGCAACCGCCGAGCTCTTGCGGCGTATATGT
ACGACAAGCGAAAAGGGCCGCTCTCTTTGCCGACAACACGCGCATGGAGCCCGAATGTGACAGACGATGCGATCC
CAACAGAGCCCTGCCATGATTTGGAATCAGACAAGCTTGTTCCCTTGATTCCTGATCCCGCGCCGCCACTGCGTCTA
GACCAAGGCGATCGACGAGAGGTGGTGTATGTTACAGTGCCCATCCTGGAGAAGTACGGGATTGTGCCGATGGG
CTTTATGAACAGCTCGACATGGCGCCCCTATGGCCAGCGTGGTCCTGGTCGTCAGCCGCTACTACACCGTTTATCG
CATGCCAACTCCACGAGTCTCGAGGACTGGCAACGCTACGATGTGTACGACCCAGGCCATGAGCTTGTGGTGCCG
ACGCACCCTAGCAAGCCTGTCGTGTTTGAGCTGGTGATCAACAACCAGGACGACTCGCCACACCCTTTCCATCTGC
ATGGCCACAAGTTCTGGGTCATGCAAACAGGCGAAATGGATCCTCGCTTTGGTGGCTACGACTACTATGAAGATCT
GGGTCAGCAGTACCAACTGGACCGGCTCATGAAGCGCGACACGGTAGTCGTCCCGATGATGGGCCATGCTGTCAT
CCGATGGGTGGCGGACAACCCTGGTGTATGGGCTTTCCACTGCCATATGCTGGTCCACCTCGCGAGCGGTATGGC
CATGGCCATTGTCGAGCAGGCGGCAGTCCTCCAAGCACAACCTCCAGTACCGCTTACATGTACGTAA

>M. Caprae LAC1

ATGGATCCTAACTACAATATCTCGGAGCCACCTCGAGAGCGGCATTTCGACTGGACCGTGCAGCGAAAAGTTGTG
GCGCCTGACGGCGTGCCTCGTTTGATGTATACCATCAATGGCCAATTCCCAGGGCCTATTATGGAAGTGACAGAA
GGCGATACCGTTGTTGTGCGCGTACGAAACCATATTTATGATGACTATCAAGTGCCTGAGCCTCCTATTACTTCGCG
CCTCCATGATGTATTCCCCGAGGGCACAGACCGCAAGTTTTCTTTTCATTGGCATGGACTGTCTATGCGCGGACAA
CAGGTGATGGATGGCGCATCTTCGTTTACGTCCTGCCCGCTGAAACCAGGGGACGAGACTGTGTATCGATTCACC
GTATATCCGGAAGATGTGGGTACACACTGGTACCATTCCCATGTCGGCACATCTCGTGCGGATGGCTTGTGGGGG
GTGTTGATTGTCCACGCTCGCGAAGATGAGCGCGCCCTTTTCCAAAAGCATCCACTAGGTTCGAAAGTGCACTGGG
ATGATGAAATCGCTGTGGCTCTTGGTGATCATTTCCATGTCATGAGCCCAGAGAGTCTGGCTGAATATGTTTCTCTC
CTTTTGTCCAATGCAGAGCCCGTGCCCGACAGTGGTCTCATTAATGGAAAGCATGTTTTCAGCTGCTACATGAGCA
AAAAAGAGGATGTCCCGTGCCCTGCTGGGGACGTCGACGAAGTGGGTAATTATGAAACCTTCCATCTCAACCCAC
AACGCTCCTACCGCTTGCGTCTGGTCAATGTAGGATCTGTGGCTGACGTTGTGTTTAGTGTGGATGGACACACGAT
GACAGTGATCGAGGCGGATGGCACGCTGGTGGAACCCATGACCGTGCATCGTATTCCCATTTCACCGGGTCAACG
ATACTCTGTCATTTTGCATCGCGAACCGCTAAGCAAAGTCAAACGTGTGTGGATGCGGGCAGAAATGACAGCCGA
GTGTTTCAAGTACCTGAATCCTGTGATGGATCCCATGGTCAAGGCCATCGTGGCGTACGATGAAACTGCTTCAGAT
CAAGACCACTGGCTTTCGCCGCTGAAGATGCGGAGCTACCGTCGCATTATATCGGAGCGGATGCTGTCACGGCGC
TGGGAATGGGCAATGCGGCCCTCCTCCAAGGCATGGAGCCCCAATGTGACCGATCTGGGCATCCCTGAGGAGCCT
TGCCATGACTTGGAGCCAGGGACCCTTATACCGCTGGTACCCGATCCAGCACCTAAGCTTGATATAAGCCGTGGA
GACCGCCAGGTCTATGTCAATATTGAGGTACTGAACCGCCACAAGTATCTTAAAGCACCCATGGCATTCCTCAATA
TGTCTACGTGGCGTCCGTATGGCCTCCGTGGTCCCTCCAAACAACCACTGTTGCATCGAATTTCGCACTCAAATCTA
TCGTCGCAGGAAGAATGGGAGCAGACTGGGCTTGTGAACCGCGAACACGAACTGGTGGTTAGTACGCATCCAAG
TAAGCCAGTCGTGTTTGAATTGGTGCTATTCAACTACGACGATGGGCCTCATCCTTTCCATCTGCATGGCCATAAAT
TTTGGGTGCTCCATACGGGTGAAATGGAACGTCTGCGTTTCCAGTACACCAAGGAGGTGGAGGAGTCCTTTGATC
TTGCACGCGCTATGAAGCGTGACACGGTCGTGGTGCCCATGCTGGGACATGCTGTGATTCGATGGGTGGCTGACA
ACCCAGGCGTATGGGCCTTCCACTGTCACATGAATGTACATCTAGCAAGTGGCATGGCTATGGCCTTCGTCGAGCA
GCCCGAGGTCCTGCAGGCTCGTCCACCGGTGCCACCCACATGTCAATAG

>M. Caprae LAC2

ATGGATCCCAACTACAACACCGCGGAGCCCCCTCGTGAGCGTGTTTTCGACTGGACAGTGGAGCGCGCAGAGCGG
GCGCCTGACGGCATTTCGCGGTTAATGTACACGATCAATGGCCAGTTTCCAGGTCCCATTATTGAAGCTACAGAGG
GCGATACAGTGGTGGTCCGTGTGAGGAACCACATTTATGACAATTATACCATGCCACCTCCGCCTCTGTCGTCCCA
GCGAAATGATGTGCATCCTGAGGGGACGGACCGTAAATTCACTTTGCACTGGCATGGCCTATCGATGCGGAACAC
GCAAGCCATGGATGGAGCGTCAGCTTTTACTTCTTGTGCCTTGACTCCTGGTGAAGAGCGCGAGTACCGCTTCCTC
GTACACCCGGAAGATGTGGGGACACATTGGTATCATTCGCATGTCGGGACGTCACGAGCTGATGGATTGTGGGG
CATGTTTATCGTCCATTCACGTACCGATGAGCGCAAGGAGCTTGCGATGCGCTCTCCTGACTCGAATACCCACTGG
GATGAGGAAGTAGCCATTGCGATCGGAGACCACTTCCACCAATTTGGACCCATCAGCCTTGGCTTTTATGTCTCGC
GCTGGCTCCAGAAGGCAGAGCCCGTGCCCGAAAATGCACTTATTAATGGTAAGCATGTATTTTCTTGCAACCACAG
CCGACTTGTTGGTGTGCCTTGCCCTGCAGGCGACGTGGAGCAGGTGGGCGAATATTCTACCTTCAGTCTAGACCCC
AAGCGCGCCTACCGCCTCCGGCTTGTCAATGTCGGTGCACTTGCCGACATTACTTTCAGCGTCGATGGTCATACCAT
GACGGTCATTGAGGCAGATGGCACATTGGTCGAGCCTGTGCGTGTCCACCGCATACCCATTGCTCCTGGCCAACG
GTATTCAGTTATTTTGCACCGCGAGCCCAAAACTGAAGACTCCCGCGTCTGGATGCGGGCTGACATGTCGGCAGA
GTGCTTTCAGTACACCAATCCTGTGATGGAGCTCAGTGCCAAGGCTATTATTGCTTACGATACAAGGCAGAGCCAC
GATGCGAGCAAACATGATCTCGAGTCTTTGCTGCGTAGGAACAGCTATAAGCGTGGTCTGGCAGACAGGATCTGG
CATGGCAGCTGGTCCTGGAGCCGATTGCCGAACACCCTGCCATGGAACCCTAATGTCACGGACGCCATTATTCCGG
AAGAGCCATGCCATGACTTGGAACCGGATACTCTTGTTCCTCTTATCCCTGATCCTGCGCCGCCACTCCGGCTCGAC
CAGGGAGATCACCGTGAGTTTGTGTTTGTCACGGCTCCTATCTTGGAGCGCTACGGTGTTGTGCCTATGGGCTTCA
TGAATAACTCGACGTGGCGCCCCTACGGACATCGTGGTCGTGAGCGCCAGCCGCTGCTGCACCGAATCTCTTATGC
AAACACTTCCACTGTGCAAGGCTGGCAAGAACAGGATATCATCGAACCCCTGCACGAGCTCGTGGCAAGCCCGCA
TCCCAGCCGGCCGGTTGTTATGGAATTGGTTATCAACAACCAGGACGATTCTCCACACCCATTCCACTTGCATGGCC
ACAAATTCTGGGTAATGGAGACAGGAGAGGCCGACCCTGAATATGGAGGATTCGATTACTACGAGAATGTGGGA
CAAGTCTACACTCTGAACCGGAAGATGAAACGAGACACAGTGGTCGTGCCCATGATGGGACATGCAGTTATCCGC
TGGGTGGCTGATAACCCAGGCGTGTGGGCCTTCCATTGCCACATGCTAGTCCACTTGGCTAGTGGCATGGCCATG
GCCATTGTTGAGCAGCCCGAAGTGCTGCAAGCGCGTCCGCCAGTTGAGCGCGTTTGTTCGTAG

>M. Cuniculi LAC1

ATGAAGAGCATTGCCACAGGGCTTCTTATATTTGCCAGTGTGGCCTATGCGGCTGTGGTAGAGTACAACTTTACCG
TCGGGTGGCACAATGCTGCTCCAGATGGCGTGGAGAAGCGCATGAACCTTGTCAATGGACTATTCCCTGGTCCTG
AAGTGCGTGCCAATGTCGGAGACACTGTTCGGATCCATATGAAGAATGATCTCGGCGACAACGGTACCTCTATTCA
CTTTCATGGAATCAACCAAAAGGGCACACCATTCAGTGATGGCGTCCCTGGTGTCACTCAGTGGTTAACCAAGCCC
GGAGATACGTACACCTATGAGTTTGTACCAGACGAGGCCGGCTCATCGTTCTGGCACTCACACATGGGTATCCAGT
ACCCTCTTGGCATGGTCGGATCCATTATTGTTGACGAAAAGAAGGGTGCCTGGGAGTATGATGATGAGCATGTCG
TAATTCTTACCGATCACTTTGACCTTTCCGAGTGGACACTTCTTGCTCGTGCCATGCAGCCCAATATGGACGATCCA
TTGGTCGACAATCTGCTTATCTGCAGCAAGCCTGGTTCGTGCAACAAGAACCAGACGCAAAAGTTCAACTTTGAGG
CTGGCAAGACGTACCGCCTTCGTGTGATCAACATGAGTGCCAACTCGTACTTCCGCTTTTCTGTCGATGACCATGAC
GTGCAAATCATCCAGACTGACTTTGGACAAACCAACGGGTCATATGCCAAGTGGGTCCCTCTTGCGCCGGCCCAGC
GCCTTGATCTGTTGTTGCACACCAAGAAGGAACAAAAGGAGAATGTCTGGATTCGTGCTGTGAGTATGACCAAGT
GCATGGGCGAACTTAAGCATGGCGTCAGCGATGAGGTCAGGGCCGTGGGTGTATTTGTCGACAAAAGCGTTACC
CCATCAACCAAGGCACACGAGTTCGAAAGCAGGAAGAAATGCTATGTGCTTGAACCGCACCAGTATGCTCCTCGT
ACAAAGTCACCTGCACACCGCAAGCCTATCCAAACGATCCGTCTGGAAGGTAACGTAAGCAAGAACAAACAGCAT
ATCCTGCTTGGCACACTGAATAATATTACGTTTACGGACAAGAACAACGCTGTCAACCCTACGCTGTTTGAACTGC
AAGCTGGCAAGAATGCGTCCGAGTTTGCCAGGTATCGCAACACGTATGTCATCCCCAAGAAAGGTCCTATTCATGT
TCTATTTGACGGTATTCAAGAGGAGGATCCTCACCCGTACCACATGCATGGACATACTTTCAAGCTGCTTGGTATC
GTTCCATCGCCATTCGACCCCAAGAACACAACTGGTCTGAACTTTGACGCTCCTGCGCATCTGGACACTGTTCTCGT
TCCTGGCAACCACACGGCTGTTTTGGAGATCATGGCCGATAACCCTGGTTCCTGGATGGTTCACTGCCATATGGAC
TGGCACCTTGCGCGAGGATTTGCCGCACAGCTGGTGGAGCTTCCGAACCTGTTTTTCAGCAAGTATGGCCGTAACT
CCACCGAGAGCACTTGAGTGACGCCTTTCTAGTTTGCATATTAA

>M. Cuniculi LAC2

ATGTGCACGGACGTCTTGTCGGGATTTCGCACTCGGTGCCGAGCTTTGGCGTGCGCGAAGACCACGGCCCTGTGC
GCCTTGACCCCCGCTTTGATACAAGGGCGCCGCCGCAGGAGCGTTTCTACGAGATGAACGTGACACGCAAGCACA
TTGCCCCCGACGGCATCCCCCGTGTGATGTACGCCATCAACGACAACTTCCCCGGCCCGACTATTGAGGCGAACGT
CGGCGACACTGTGGTGGTCGTGCTGCGCAACCATATTGACGACGACTTTACGTTCCGCACGGACCTCATGACGAG
TCGCTTCCAGCACGTGCATCCGCCCGGCTACGACCGCAAGGTGCTTATTCACTGGCACGGACTGTCGATGCGCGAC
ACGCAGGTGATGGACGGTGGCGGCGGATTCACGAGCTGCCCTCTGCAGCCCGGGCAGGAGTTCACGTACCGCTT
CAAGCTGCACCCCGAGGACGTGGGCACGCACTGGTACCACTCGCACTCGGGCATGTCGCGTGCGGACGGCCTGT
GGGGCATGCTCGTCGTGCATGCGCGTCAAGACGAGAGCCTGCTGCTCAAGCAGTATGCGCCGCGGGTGGCGCAG
AACGTGTCGCTGGAGTGGGACGAGGAGGTGCCTGTGGCGATCGGGGACCACTTTCACAAGCCGGGCCCCGAGTT
TATGGCGTGGTACGTGTCCAAGTGGTCGATGCAGGGTGCGGAGCCCGTGCCGGACAATGGCCTGTTCAACGGAC
GGAACCGGTTCAGCTGCGAGCACAGCCGGATGGTGGATGTGCCGTGTCCCGCGGACCGCTTTGGCAAGGAGGTC
GTCGGCGACCACCAGCACTTTCGGCTGCGGGACGACAAGCGGTACCGGCTGCGCATCGTGAACGTGGGTGCGAT
CTCGGACATGGTGTTTTCCGTGGACGGGCACACGCTGACGGTGATCGAGGCGGACGGCACGCTGGTCGAGCCGA
TCACGGTGCACCGGGTGCCGATTGCGCCTGGGCAGCGGTACTCGGTGCTGCTGAACCGTGTGGAGACGGAGGAC
GGCGCGATGCCGTCGCGCGTGTGGATGCGTGCGGAGATGGCGCCCGAGTGCTTCCAGTATGCGAACCCCCATCTG
GACTACACGACGCGAGCGGTGCTGTCGTACTCGCCGGGCGACAAGGTGGCTGGACAGGGCGGATGGTTTGCGCC
GCTGCGCATGCGCATGCGCGGAGCTCGCGGACGGCTCGCGGCGCGCACGACGGCGATCCGCGAGTCACGTGGCC
TGGTCAATGTGCTCCCCCAGACGCGTCCGTGGAGTGCGAACATCAAGGACGCGGAGGTGCCCGAGGAGGCGTGC
CACGACCTCGAGACTGGGTACCTGCTGCCGCTGGTGCCTGACCCGGCGCCCGAGCTGGACTTTGAGGCGGGCGAC
CAGCGGGTGATCGTGTATGTGACGGTGCCCAAGCTGGAGCGGTGGGGCGTCGTGCCCATGTCGTACATGAACTC
GTCGACGTGGCGGCCGGAGGGCGGCTTCACGGGCAAGCCGCCGCTGCTGCACCGGATCTCGCACGCAAACACGT
CGACGGCAAGAGAGTGGGAGGAGACTGGCGTGGTGAACCGCGAGTGGGAGCTGGTGGCGAGTCCGCACCCCAG
CCGGCCGGTGACGATTGAGCTGATTGTGAACAACCGCGACGACTCGCCGCATCCGTTCCACCTGCACGGACACAA
GTTCTGGGTGCTGGACTCGTACGAGATTGACATGAAGTACGGCGGCTATGGAGACTGGGACGGCGAGGAGAGCA
AGATGGACGTGCGGCGCGTGATGAAGCGCGACACGGTGGTGGTGCCGATGCGCGGGTACATGCGGCTGCGCTG
GCGCGCAGACAACCCGGGCGTGTGGGCGTTCCACTGCCACATGCTGGTGCATCTCGCGTCGGGCATGGCGATGTC
GTTTGTGGAAATGCCAGAGGTGCTGCAGGCGAGTCCTGGGGTGCCGGCACAGTGTAA

>*M. equina LAC1*

ATGGATCCCAACTACAACAGCTCAGCGCCTCCGCAGGAACGACATTTTGACTGGACAGTGGAGCGAAAAATAGTA
GCACCGGATGGTGTGCCCCGCTTGATGTATACCATTAACGGCCAGTTTCCTGGCCCCATTATGGAGGTGACGGAA
GGGGATGTTGTTGTGGTGCGAGTCCGGAATCAAATCTTTGATGACTATGAGGTACCTGAGCCTCCTATCACTTCGC
GCCTTGAAGATGTATTTGCCGAGGGCACTGACCGTAAATTTTCCTTTCACTGGCATGGTCTCTCCATGCGGGGACA
GCAAGTGATGGACGGGGCGGCTTCCTTTACATCCTGTCCGCTCAAGCCAGGCAATGAGACCGTGTATCGATTCACC
GTGCATCCTGAGGATGTCGGTACGCACTGGTATCATTCCCATGTGGGAACATCGCGTGCGGACGGCTTGTGGGGT
ATGCTGATTGTCCATGCTCGCACCGATGAGCGTGCCCTATTCCAAAAGCATAGGCCGACTGCGAACGTGCACTGG
GACGACGACATTGCCGTGGCGTTGGGAGATCATTTTCACGTCATGAGTCCGGAAAGTCTCGCAGAATACGTCTCCT
TGCTTTTGTCCAACGCAGAGCCCGTGCCAGACAGTGGCCTCATTAATGGAAAACACGTATTCAGCTGCTATATGAG
CAGGATGGAGGATGTTCCGTGTCCTGCTGGCGATGTCGACGAAGTGGGCGAGTATGAAACATTCCACCTGAAGCC
TCAGCGCAGCTACCGCCTGCGTCTCGTCAATGTGGGGTCGGTCGCCGACGTGATTTTCAGCGTGGACGGTCACAC
AATGACTGTGATCGAGGCGGATGGAACCTTGGTGGAACCCATGAACGTACATCGTATTCCCATTGCGCCTGGCCA
GCGCTACTCTGTCATCTTGCACCGTGAACCCTCAAGCAAGGCGGACCGCGTGTGGATGCGAGCGGAGATGTCGGC
AGAGTGCTTCAAGTACTTGAATCCCGTGATGGATCCTATGGTCAAAGCCATCGTGGCGTACGACGAGAGTGCCGA
AGACGATCACCACTGGCTATCGCCCTTGAGGATGCGAAGCTACCGCCGTGCTATTTCGGAGAGCATCTGGGCACG
GCGTTGGGGCTGGACGCTGCGTCCTTCGTCCCAGCCATGGAGCCCCAACGTGACCGATGTGGGCATTCCCGACGA
GCCATGCCATGATCTCGAGCCAGGGACGCTCGTGCCCCTAATCTCTGACCCCGCACCCAAGCTGGACGTGTCCCAA
GGCGACCGCCAAGTCTATGTGAATATCGAGGTGCTGAACCGCCACAAGTATTTCAAGGCACCCATGGCATTTATG
AACATGTCGACGTGGCGCCCCTACGGTCGCCTTGGGCCAGCCAAGCAGCCTCTATTGCATCGCATCTCGCACTCAA
ACACCTCCTCGCCTCAAGAATGGGAGCAGATGGGGCTTGTGAACCGCGAGCATGAGCTGGTGGTAAGCACACATC
CAAGCAAGCCCGTTGTTTTCGAGCTGGTGCTCTTTAATTACGACGATGGCCCTCATCCCTTCCATTTGCATGGTCAC
AAGTTCTGGGTTCTTCACATTGGAGAAATGGAGCGGGTACGATTCAAGTACACCAAGGACGTGGAAGATTCCTTC
GACCTGACACGCGCTATGAAGCGCGACACCGTTGTGGTGCCCATGTTGGGTCATGCGGTCATTCGATGGGTGGCC
GACAATCCCGGTGTATGGGCTTTCCATTGTCACATGAATGTCCATCTCGCCAGTGGTATGGCGATGGCCTTTGTCG
AGCAGCCCGAGGTCCTGCAGTCCCATCCTCCTGTGCCGCCCACCTGTCAGTAG

> *M. equina LAC2*

ATGAATCCCAACTACAATACCGCGGAGCCACCTCGTGAGCGTGTGTTTGATTGGACAGTGGAGCGTGCAGAGCGG
TCGCCTGACGGTATATCGCGGTTAATGTACACTATTAATGGCCAGTTCCCGGGTCCCATCATTGAAGCTACAGAAG
GTGATACTGTGGTGGTTCGTGTGAGAAACCACATCTATGATAATTACACCATGCCATTGCCGCCTCTGACGTCCCA
ACGAAATGATGTGCATCCGGAGGGGACGGACCGTAAATTTACGTTCCACTGGCATGGCCTGTCCATGCGGAACAC
GCAGGTCATGGATGGTGCATCAGCTTTTACTTCTTGTGCCCTGCTCCCTGGAGAAGAGAGAGAGTACCGCTTCCTT
GTGCATCCGGAAGATGTTGGGACGCATTGGTATCACTCGCACGTCGGCACATCCCGAGCTGACGGCTTGTGGGGC
ATGTTTATCGTCCACTCGCGTACCGATGAGCGTAAGGAACTTGCGAAACGTTCTCCAAAGTCGAATACCCATTGGG
ATGAGGAAGTGGCCATTGCATTGGGCGATCACTTCCATCAATTTGGACCCATTAACCTCGGCTTTTACGTATCACG
ATGGCTTCAGAAGGCAGAGCCTGTTCCTGAAAATGCCCTAATCAATGGCAAGCATGTGTTTTCTTGCAACCACAGC
CGTCTTGTTGGTGTACCTTGCCCTGCAGGCGATACCGACCAAGTGGGCGAGTATTCTACCTTTAGTCTGGATCCAG
AGCGGGCCTACCGTCTTCGACTCGTGAATGTCGGTGCACTCGCTGACATTACTTTCAGCGTTGATGGTCATACCAT
GACGGTTATTGAGGCGGATGGCACACTGGTCGAGCCTGTGCATGTCCACCGCATTCCTATCGCTCCTGGCCAACGA
TACTCGGTCATTCTGCACCGGGAGCCCAAGAACATGGACTCCCGCGTCTGGATGCGTGCTGAAATGTGGGCAGAG
TGCTTCCAGTACACTAATCCTGTGATGGAACTGAGTGCCAAGGCTATCATTGCATACGATACAAGACAGAGTCAAG
TTGAGAGCAAGCATGATTGGGAGTCACTGTTGCGCATGCGCAGCTATAAGCGTGGCTTGGTGGACAAGATTTGGC
ATGGTAGCTGGGCTTGGAGCCAGTTGCCGACCACACTTCCTTGGAACCCCAATGTCACGGACGCGATTATTCCGAA
TGAGCCATGCCACGACTTGGAACCGGATACGCTTGTCCCTCTTATCCCTGATCCTGCGCCTCCGCTCCGGCTTGACC
AAGGCGACCACCGTGAGTTTGTGTTTGTCACGGCTCCTGTCCTGGAGCGGTACGGCGTCGTGCCCATGGGTTTCAT
GAATGGATCGACTTGGCGCCCCTATGGACATCGTGGACGTGAGCGCCAACCCCTGCTGCACCGCATCTCATATGCA
AACACATCTACTGTGCAAGGCTGGCGTGAGCAAGATCTCTTTGAACCCGAGCATGAGCTTGTGGCGAGTCCACAT
CCCAGCCGGCCGGTGGTTATGGAAATGGTTATCAACAACCAAGACGATTCCCCACACCCATTCCACTTGCATGGTC
ACAAGTTTTGGGTGATGGAGACTGGGGAGGCGGACCCTGAATATGGAGGATTCGACTACTACGAAGATGTGGGG
CAAGTCTACAATTTGAGCCGGAAGATGAAACGGGACACTGTGATCGTGCCCATGATGGGACATGCAGTTATCCGC
TGGGTGGCTGACAACCCAGGTGTGTGGGCACTGCATTGCCATATGCTAGTACACTTGGCCAGTGGCATGGCCATG
GCCATTGTTGAGCAGCCGGAGATCCTGCAAGCTCGACCGCCTGTATCTCGTACATGTTCGTAG

>M. dermatis LAC1

ATGGATCCTAACTACAATAGCTCAGCACCGCCTCAGGAGCGTCATTTCGACTGGACAGTGGAACGAAAGGTGGTC
GCTCCTGACGGCGTGCCTCGTTTGATGTATACCATCAACGGCCAGTTCCCAGGACCTATCATGGAAGTGACAGAAG
GCGATACCGTTGTTGTGCGAGTACGAAACCATATTTTTGACGATTATCAAGTGCCTGAGCCTCCTATTACTTCTCGT
CTCTATGATGTATTCACAGAGGGCACAGACCGCAAGTTTTCCTTTCATTGGCATGGACTATCCATGCGCGGACAAC
AAGTGATGGATGGTGCATCTTCTTTCACATCCTGTCCACTTAATCCAGGAAATGAGACGGTGTACCGGTTTATTGT
GTATCGGGAAGATGTGGGTACACACTGGTATCATTCCCATGTCGGCACATCTCGTGCGGATGGATTGTGGGGAAT
GTTAATTGTCCACGCTCGCGAGGATGAGCGCATCCTATTCCAAGAGCGCGTATCAGGCTCGAATGTGCACTGGGA
TGACGAAATCGCTGTGGCTCTTGGTGATCATTTCCATGTCATGAGCCCGGAAAACCTAGCCGAATATGTTTCCCTCC
TTTTGTCTAATGCAGAGCCCGTGCCAGACAGTGGTCTCATCAATGGGAAGCATGTATTCAGCTGTTACATGAGCAG
AAAAGAGGACGTCCCGTGTCCTGCTGGCGATGTCAACGAAGTAGGCGAGTATGAAACCTTCCACCTCAATCCACA
GCGCGCCTACCGTTTGCGCCTGGTCAATGTGGGATCTGTGGCTGATGTCGTGTTCAGCGTGGATGGACACACGAT
GACCGTGATTGAGGCGGACGGTACGCTGGTGGAACCCATGACCGTGCACCGTATTCCCATCTCACCGGGTCAACG
ATACTCTGTCATCTTGCATCGAGAACCGCTCAGCAAAGTTGACCGTGTGTGGATGCGGGCAGAAATGACTGCCGA
GTGTTTCAAGTACCTGAATCCTGTAATGGATCCCATGGTCAAGGCAATTGTGGCGTACGATGAGAGTGTTCCAGAC
CAGGACCACTGGCTTTCGCCGCTTAGGATGCGGAGTTACCGTCGCATTATATCAGAGCGTATCCTGTCACGTCGCT
GGGGCTGGACACTGCGGCCTTCCTCCGAGGCATGGAATCCCAATGTGACCGATGTGGGCATCCCTGAGGAGCCTT
GCCATGACCTGGAGCCAGGGACGCTTGTGCCACTGGTGCCCGATCCAGCACCTAAGCTTGATATAAGCCGTGGGG
ACCGCCAGGAATATGTCAATATCGAAGTGCTGAACCGCCACAAGTATTTTAAAGCACCCATGGCATTCCTTAATAT
GTCTACGTGGCGTCCGTACGGCCTCCTTGGACCATCCAAACAACCACTACTGCATCGTATCTCACACTCGAATCTAT
CCTCGCCCGAAGAATGGGAGCAGACTGGACTTGTGAACCGCGAACACGAGCTAGTGGTAAGCACGCATCCAAGC
AAGCCGGTTGTGTTCGAATTGGTCTTATTCAATTACGACGATGGGCCACATCCTTTCCATCTGCATGGCCACAAATT
CTGGGTGCTGCACACGGGCGAAATGGAGCGCCTTCGCTTTCAATACACCAAGGAGGTGGAGGAGTCTTTCGACCT
GGAACGCGCTATGAAGCGTGACACGGTAGTGGTGCCTATGTTAGGACATGCTGTGATTCGATGGGTGGCAGACA
ACCCTGGCGTATGGGCTTTCCACTGTCACATGAATGTGCATCTTGCAAGTGGCATGGCCATGGCCTTTGTCGAGCA
GCCACAGATCCTTCAGGCTCGCCCTCCGGTGCCGTCCACCTGTCAATAG

> *M. dermatis LAC2*

ATGAACCCCAACTACAATTCCACGGAGCCGCCTCGTGAACGTATTTTCGACTGGACAGTGGAGCGCGCAGAGCGG
TCGCCCGACGGTATATCACGGCTAATGTACACGATCAATGGCCAGTTCCCGGGCCCCATGATTGAAGCTACGGAA
GGTGATACAGTGGTGGTTCGTGTGAGGAATCATATCTATGACAATTATACGATGCCGCCGCCGCCCCTGTCGTCC
CAGCGAAATGATGTGCATCCTGAGGGGACAGACCGTAAGTTTACTCTCCACTGGCATGGCCTATCCATGCGGAAT
ACGCAGGTCATGGACGGTGCATCAGCCTTTACTTCTTGTGCCTTGGTCCCTGGCGAAGAGCGAGAGTACCGCTTCC
TCGTGCATCCGGAAGATGTGGGGACACATTGGTATCATTCACATGTCGGAACATCACGAGCTGACGGATTGTGGG
GCCTGTTTATTGTTCACTCGCGCACTGATGAGCGCAAGGAATTTGCGACACGTTCCCCAGAAGCGGATACCCATTG
GGATGAGGAAGTGGCCATTGCGCTTGGCGACCACTTCCACCAATTTGGCCCCATTAACCTTGGCTTGTACGTCTCG
CGCTGGCTCCAGAAGGCGGAACCTGTGCCCGAAAATGCATTGATCAATGGCAAGCATGTGTTTTCTTGCAACCACA
GTCGTCTTGTTGGCGTGCCTTGCCCTGCAGGCGATGCCGAACAAGTGGGTGAGTATTCTACTTTTAGTCTGGACCC
AGAGCGTGCCTACCGCCTCCGGCTCGTCAATGTTGGTGCACTTGCCGACATTATTTTCAGCGTCGATGGCCATACC
ATGACGGTCATTGAGGCAGATGGCACACTGGTTGAACCTGTGCGGGTCCACCGCATACCCATTGCTCCCGGTCAA
CGATACTCAGTCATTTTGCACCGCGAGCCCAAGAGCAAGGACTCTCGGGTCTGGATGCGGGCTGACATGTCGGCA
GACTGCTTCCAGTACACTAACCCTGTGATGGAGCTCGGTGCCAAGGCCGTCATTGCATACGATACAAGACAGAGC
CAAGATACGAGCAAGCATGATTTGGAGTCCCTGCTTCGTATGCGAAGCTATAAGCGTGGTTTGACAGACAAAATC
TGGCATGGTAGCTGGTCCTGGAGCCAGTTACCGACCACGCTGCCATGGAACCCTAATGTTACGGACGCAATTAT
TCCAGAGGAGCCGTGCCATGACTTGGAACCAGACACACTTGTTCCACTTATCCCTGATCCTGCACCACCGCTCCGG
CGCGACCAAGGCGACCACCGCGAGTATGTGTTTGTCACGGCTCCTGTCTTGGAGCGTTACGGCGTTGTGCCTATG
GGCTTCATGAATAGCTCGACATGGCGCCCCTATGGACATCGTGGACGTGAACGCCAGCCCCTGCTACACCGCATCT
CTCATGCAAACACTTCGTCTGTGCAAGGCTGGCGAGAGCAGGATATCATCGAACCCCTACATGAGCTCGTGGCGA
GTCCGCATCCTAGCCGGCCAGTTGTTATGGAATTGGTCATTAACAACCAGGACGATTCTCCTCACCCATTCCATTTG
CATGGTCACAAGTTCTGGGTTATGGAGACAGGAGAGGCGGACCCTGACTATGGTGGATTCGATTACTACGAGGAT
GTGGGACAAGTCTACAATCTAAGCCGGAAAATGAAACGAGACACGGTGATTGTGCCTATGATGGGACATGCAGTC
ATCCGCTGGGTGGCCGATAACCCGGGCGTGTGGGCCCTGCATTGCCACATGCTAGTCCACTTGGTCAGTGGCATG
GCCATGGCCATTGTGGAGCAGCCCGAGATCTTGCAGGCGCGACCGCCGGTGCCGCCGATTTGTTCATAG

>*M. nana LAC1*

ATGGACCCCCATTACAATCATTCTGCTCCACCACAGGAGCGGCATTTCGACTTGACTGTGGAGCGTAAAATGGTGG
CACCGGATGGTGTGCCGCGACTGATGTACACCATCAACGGCCAGTTTCCTGGGCCTACGATCGAGGCCACCGAAG
GGGATACGATCGTAGTGCGGGTGCGCAATCATATCTTCGACGACTATGTGGTACCTGAGCCTCCCATCACGTCCCG
CCTTTATGATGTGTTTTCGGAAGGCACCGATCGCAAGGTCGCGCTCCACTGGCATGGACTGTCTATGCGGGGACA
GCAAGTGATGGATGGGGCCGCCTCGTTTACGTCCTGCCCAATTCTCCCCGGGAATGAAACAGTGTATCGGTTTACT
GTATTGCCTGAGGATGTTGGCACCCATTGGTACCACTCGCATGTTAGCACTTTGCGCACTGACGGACTGTGGGGCA
TGCTGATTGTCCATGCACGGAAGGACGAGCGTTCTTTATTCCAGGAGCGTGCGCCGAATGCTCGTATTCACTGGGA
CGAAGAAATTGCTGTGGCACTGGGCGATCACTTCCATGTCATGAGTCCTGAGAGCCTCGCCGAATATGTATCCCTC
CTATTGTCGAATGCGGAGCCCGTGCCGGACAATGGACTTATCAATGGGAAGCATATATTCAGCTGCGCAACGAGC
AGAAAAGAAGATGTTCCATGCCCTGCAGGTGATGTCGACATGGCCGGGGAGTACGCGACTTTTCATCTGAACCCT
CAGCGCACATACCGTTTGCGCCTAGCCAATGTTGGTGCATTGGCTGATGTCACGTTCAGTGTTGATGGTCATACAA
TGACAGTGATTGAAGCTGATGGGACACTGGTGGAGCCCATGACCGTACATCGTATCCCGATTGCTCCAGGTCAGC
GATACTCTGTCATTCTGCATCGCGAGCCATCCAGCAAAAGAGATCGGGTGTGGATGCGGTCGGAGATGAGCGGC
GAATGCTTCAAATACATGAATCCTGTGATGGACCCTATGGTGAAGGCGATCGTTGACTACGATGATGACGATCTCC
ACTGGATGTCGCCCTTGCGCTTGCGGAGCTACCGCCGCGCATCGGAGCGCCTATGGTCGAAGCGCTGGGGCTGGA
CAGTATGGCCCACGTCGCAGCCATGGGGCGACAATGTGACCGATGCCGGCATTCCAGAGGAGCCGTGCCATGACT
TGGAGCCTGGTGTACTAGCGCCGCTCATCCCGGACCCACCGCCTGCGCTCGATTTCAGCCGGGGCGATCGCCGCG
AAATTATCAATATCGAAGTGCTGAACCGCCACAAGTACATGAAGGCGCCCATGGCTTTCCTGAATATGTCTACGTG
GCGTCCATACGGCCATCGCGGATCATCTGTGCAGCCACTGCTTTATCGTATTTCTCATTCGAATGTGTCGACTCCCG
AAGAATGGGAGCGGATGGGGCTGGTCAACCGCGAGCATGAGCTGGTGGTAACAACGCATCCCAACCAACCAACG
GTGTTTGAGTTGGTGTTGTACAATTACGACGATGGTCCCCATCCATTTCATTTGCACGGCCACAAATTCTGGGTGCT
GCATACGGGCGAAATGGAGCGGCTGGCCTTTCAGTATACGAAAGAAGTGGAGGCGTCTTGGGACCTGTCGCGCG
TCATCAAGCGCGACACGGCTGTCGTGCCTATGCTTGGGCACGCAGTCATTCGGTGGGTCGCTGATAACCCTGGCG
TATGGGCTTTTCATTGCCACATGGATGTGCACCTCGCTAGTGGCATGGCCATGGCATTCGCCGAGCAGCCCGAAGT
GTTGCAGGCGCACCCTCCGGTCCCCGCTACCTGTTCATAG

>M. nana *LAC2*

ATGGATCCGAAATATGACCCCACCCAGCCACCGCAAGAGCGTGTCTTTGACTGGACGGTCGAGCGTGCCGAGCTT
GCTCCGGATGGGATTCCGCGGCTCATGTACACGATCAATGGCCAGTTCCCTGGCCCGCTAATCGAGGTCACCGAA
GGTGACACGGTGGTGGTTCGTGTGCGTAACCATATGTACGATAACTATACCATGCCGCCGCCGCCCCTCTCGTCGC
AGCGCGAGGATGTGCATCCAGAAGGAACGGATAAAAAGTTTACCTTCCACTGGCATGGTTTGTCGATGCGGAACA
CCCAGGTCATGGATGGAGCATCGGCCTTTACGTCATGTGCCTTGCGACCCGGTGAGGAGCGCGAGTATCGCTTCC
TCGTACACCCCGAGGATGTAGGGACGCACTGGTACCACTCGCACGTGGGAACATCGCGGGCGGACGGGCTTTGG
GGTATGTTTATTGTACACTCTCGTTCGGACGAGCGTAAAGAGTTCCAGTCCCGCTCTCCCGATGCTGATACCCACTG
GGACGAAGAAATCACTGTGGCCTTGGGCGACCACTTCCACAAGTTTGGCCCGATCAACCTGGGCTTTTACGTCTCG
CGCTGGCTTCAAAAGGCGGAGCCGGTGCCTGAAAATGCATTGATCAATGGAAAAAATATATTCACCTGCCACCAC
AGCCGTCTCAACGACGTGCCATGCCCAGCGGGCGATGCAGACCAGGTGGGTGAATACTCTACCTTTAGTTTGCGC
CCTGATCAAGCGTACCGCCTTCGTCTCGTCAATGTCGGCGCGCTTGCAGACATTACCTTTAGCGTGGATGGACATA
CTATGACTGTGATTGAAGCGGATGGCACGCTCGTGAAGCCTGTGCGTGTTCACCGCATCCCTATCGCCCCAGGCCA
GCGATACTCGGTTATCCTGCACAAGGAGCCCAAGAGCAAGGATGCACGCTTTTGGATGCGTGCTGACATGTCACC
CGATTGTTTCCAGTACACGAACCCAGTGATGGAGCTCTCGGCCAAGGCCATTGTTGCGTACGATACTAGGCAGGA
TACCGAGAGGCAGAGCGATTGGGAGTCACTGCTGCGTATGCGCAGTTATAAGCGCAACCTGGTTCAACAGATCTG
GTATGGCCGCTTGTCTGCCAGCTTGCCGGCGACATTGCCTTGGAGTACGAATGAAACCGATCCCATTCTTCCCGAA
GAGCCATGCCATGACCTGGAGCCAGACACGCTTCTCCCCCTCATTCCTGATCCTGCGCCGCCGCTCCGGCTCGACC
AAGGGGACCATCGCGAGTATGTGTTTGTTACTGCGCCGATCCTTGAGCGATACGGCATTGTGCCGATGGGCTTCAT
GAATGGCTCTACATGGCGACCGTATGGTCACCGAGGGCGTGAGCGTCGGCCCTTGCTGCACCGCATCGCTCATGC
CAATACGACCACCGTGGAAGGCTGGCGTGAGCAGGACGTGTTCGACTCGAAGCATGAGCTTGTGGCAAGTCCAC
ACCCCAGTCGCCCGGTGGTCATGGAGATGGTGATCAACAATCAGGATGACTCCCCGCATCCCTTTCATCTGCACGG
CCACAAGTTTTGGGTGATGCAGACCGGGGAGATGGACCCCGAGTTTGGTGGTTTTAACTACTACGAGGACGTGGG
GCAAGTCTACGATCTCGACCGGAAAATGAAGCGCGACACGGTGATTGTGCCCATGTTGGGACATGCGGTGATCCG
CTGGGTGGCTGACAACCCAGGCGTATGGGCTCTGCACTGTCACATGCTGGTCCACTTGAACTCGGGCATGGCCAT
GGCGATTGTGGAGCAGCCCGCGCTCCTCCAAGCGCGTCCACCCGTGCCGCCTATTTGTTCCTAG

>M. restricta LAC1

ATGTATACCATTAATGGCCAATTTCCAGGCCCAACAATCCAGGCAACTGTGGGTGACACGGTTGTAGTCCATGTCC
GCAACCGCATCAATGATGATTACGTTGTGCCTGATCCGCCTACGACGTCCAAACTAGAAAGTGTGCACCCGAAGG
GGACAGACCGTAAATTTAGCTTGCACTGGCATGGTCTGTCCATGCGTGGATCGGACGAGATGGACGGTGCAGCTG
CTTTTACGTCATGCCCTCTTCAGCCGGGTAATGAAACTACATACCGCTTTGTCGTCCATCAGGAGGACGTGGGCAC
ACACTGGTACCACTCTCACGTCGGTACTCGCGTGCCGATGGACTTTGGGGCATGCTGATCGTGCACGCGCGTGAA
GATGAGCGCAAAGTGCTGAAAGAGCGCGCCGACGTTTGATACACATTGGGACGAAGAGATTCCTATCGCGCT
GGGTGACCACTTTCACAAAATGAGTCCTGAAAGCTTGGCAAAGTATGTATCTATCGTGTTGGGTGAAGCAGAGCC
AGTGCCAGAAAGTGGTCTCATCAACGGCCGTCACATTTTTTCGTGTGACATGGCACGCTACACTGGGGTTCCATGC
CCGGCAGGCGACAAGGATGAAGTCGGCGAGTACACCGAATTTCATCTGCGTCATGACAAGCAGTATCGCCTGCGT
CTTGTCAATGTGGGCTCGATTGCCGATATTACTTTCAGTGTAGATGGGCACACGATGACTGTCATCGAGGCTGATG
GTACACTCGTGGAGCCCATGCATGTGCATCGGATACCTATTTCTCCTGGCCAGCGGTACTCGGTCATTCTGCATCGT
GAACCGTCCATGAAGGACAGTCGTGTCTGGATGCGTGCCGAGCTGCAAGGCGAATGCTTCAAGTACATGAATCCT
GTTCTGGACCCTTTCATTAAGGCGATTGTTGTGTACGATAATGCCTCACCAAAAGATGACTGGCTGGCGCCACTTC
GTGTGCGCCAAAATTACGCCAAGCGCTTTGGGCCGTTCCGTGCCTTTGCGAAATCAGAGCGTCCGACGACGCATCC
ATGGAGTCCTGATATTCAGGATCAAGGTATTCCCACCGAGCCTTGCCATGACTTGGAACCCGGAACATTGGTCCCG
CTAATCCCTGATCCGGCACCCGAGTTCCATCCTGAGCGCGGCGACCGCCGCGAGATCATCAACATTGAAGTACTCA
TTCGCCAAAAATTCAGGGCACCCATGTCGTATATGAACCTCAGCTCGTGGCGTCCCTATGGTGCCCGTGGACCTGC
CATGCAGCCGCTTCTCCATCGTATTTCCCACTCAAACTTGACGACAGCAGAAGAGTGGGAAGCGCATGGCCTTGTA
AATCGTGAGCATGAGCTAGTGGTAAGCACACATCCTACCAAACCTATCGTCTATGATCTTGTAATTGTAAACTTGG
ACGATGGGCCGCATCCTTTCCACTTGCATGGCCACAAGTTTTGGGTGCTTCATACCGGAGAGATGGAAGTGCCAG
GCTATCGCTATAAGCCCGAGGTTGAGAAGGAGTTTGACCTCAAGCGCGCCATGAAACGCGACACGGTCGTTGTGC
CGATGATGGGCCATGCCATCATTCGCTGGGTTGCTGACAATCCAGGCGTGTGGGCTTTCCACTGCCATATGCTCGT
GCATTTGGCCAGTGGTATGGCCATGGCTATTGTGGAGCAGCCTGCCTTGTTGCAAGCCAACCCATCTGTGCCAAGC
ACTTGTCAATAA

>*M. restricta LAC2*

ATGAATCCTCGCTATGATCCGACAGAGCCTCCTCGTGAGCGCTTTTTGATTGGACAGTCGAACGTGCCGACCTTG
CGCCTGACGGCGTGACGCGTTTGATGTATACTATAAATGGCCAGTTCCCAGGCCCCATTATGCAAGTTACTGAGGG
AGATACGGTCGTGGTCAAGGTTCGCAACCATATATTTGACAACTATACCGTCCCCCCTGCACCTATGTCGTCGAAA
CTGTATGACGTGCACGCTAAGGATACGGACCGCAAGTTTACTATTCACTGGCACGGCTTGTCGATGCGAGGCATG
CAAGTCATGGATGGCGCCTCTGCATTTACGTCCTGCGCACTCACGCCAGGCGACGAGTACACATACCGCTTTGTCG
TGCAGCCAGAGGACGTGGGCACACACTGGTACCACTCTCACGTCGGTACATCGCGTGCCGATGGACTTTGGGGCA
TGCTGATCGTGCACGCACGTGAAGATGAGCGCAAAGTGCTGAAAGAGCGCGCCGACGTTTGATACACATTGG
GATGAAGAGGTGGCGATTTCTGTTGGCGATCACTTCCATGATATGGGCCCAGAGTCTTTGGCACGCTACGTCTCAC
GTTGGCTCCAAAAGGCCGAGCCTGTGCCGGAGAATGCTCTTATTAATGGAAAACACGTTTTTAGCTGTGAGCACA
GCCGCTTGTCCGGTGTGCCCTGTCCAGCTGGCGATGCTGACGAGGTGGGCGAGTACAGCTCGTTCCATTTCCGTCC
AGACCGCGCTTACCGTCTTCGATTGGTCAATGTGGGATCATTGGCAGACATCACTTTCAGTGTCGACGGACACACG
ATGACCGTCATTGAGGCGGACGGCACGCTTGTGGAGCCATTGCGGGTCCACCGCATCCCGATCGCACCCGGTCAG
CGGTACTCGGTGATTCTGCACAAAGAGCCGACTACGAAGGCAGACCGAATGTGGATGCGTGCAGAAATGTCACAT
GAATGCTTCCAGTACACGAATCCGGTGATGGAGCTCGAAAGCAAGGCCATTGTAGCATACGACGGGCGCCCACCG
GCTTGGGAGGACGACGAAACGCATCAACTTTTCCCCCTGCGTCTGCGGAGCAACCGCGCCAAAGTGTCTGAATAT
GTGTACAGCAATGGTGCGACACTAAAACTGCCCGATACGAAGGCGTGGAGCCCGAATGTTACGGATGCAGCGAT
CCCCACAGAGCCTTGTCACGATCTTGAGCCAGACACGCTTGTGCCCCTTATCCCTGATCCTGCCCCAGAACTGCGAC
TCGATCAGGGCGATAAGCGGGAGTTTATCTATGTCACTGTGCCGATTCTTGAAAAGTACGGCATTGTGCCCATGG
GCTTCATGAATGGCTCGACGTGGCGCCCCTATGGCCAGCGCGGACGCGACCGCCAACCCCTTCTCCACCGCATCTC
TCACGCAAACTCGACATCTGTGCGGGATTGGTACAGTACAGATGTCGCTGATCCTATGCATGAGCTGATTGCCAGT
CCTCATCCCAGCAAGCCTGTGGTATTTGAGCTCGTGATCAATAACCAGGACGACTCTCCTCATCCGTTCCACCTTCA
CGGGCACAAGTTTTGGGTCATGCAGACGGGCGAGATTGATCCAGCGTACGGCGGCTATGACTACTATGAAGACGT
TGGACAGGTGTATCCGCTGGATCGCCGGATGAAACGTGACACGGTCGTAGTGCCGATGATGGGCCATGCGGTCA
TCCGCTGGGTTGCTGACAATCCAGGCGTGTGGGCTTTCCACTGTCATATGCTCGTGCATCTGGCCAGTGGCATGGC
CATGGCTATTGTGGAGCAGCCTGCCTTGTTGCAAGCCAATCCCCCTGTGCCAAGCGTATGCAAGCTTTAA

>*M. slooffiae LAC2*

ATGTCGCCGGCGTTCGACACGCGCGCTCCGCCGCAGGAACGCCGCTACGAGATGGTCGTGACCCGGGGCGAGCG
CGCGCCGGACGGAATTCCGCGGCTCATGTACCTGATCAACGGCCAGTTCCCCGGTCCCACGATCGAAGCGAACGA
GGGCGACACGATAGTGGTGCACCTGCGGAACGGCATCGCGGACAACGCCACGTTCAACACCAACTTCCTCTGGAC
GCAGTACGCCAAGTCGCACAAGACCAACATGGACCGCATGGTGCTGCTGCACTGGCACGGCCTCTCGATGCGCGG
CTCGCAGACCGAGGACGGCGCCGGCGGCTTCACCAGCTGTGCGCTGCACCCCGGCGAGGAGCGCGAATACCGGT
TCAAGGTGCATCCTCGCGACGTCGGCACCCACTGGTACCACTCGCACATGGGCACGTCGCGTGCCGACGGGCTCT
GGGGCATGTTCATCGTGCACTCCCGGCGCGACGAGCGCCGGCTGAGCGGCGTGCACTGGGACACCGAGGTGCCC
GTGGCGATTGGCGACCACTACCACCGGCCGAGCCCCGAGTGGTACGCCTGGTACATTTCGCGCGGCTCCTTCGGC
GCGGAGCCCGTGCCGGACAGCGGGCTGATCAACGGGCAGCACGTGTTCAACTGCGAGCACAGCCGGCTGACCGA
GGTGCCGTGCCCCGCGCAGCAGCGCTTCGGCCGCGACCGGGTGGGCCGCTACACCGAGTTCCGCCTGGACCCCGC
CAAGCGCGCGCGGCTGCGCATCGTGAACGTCGGCGCGGTGGCGGACATGACCTTCTCCGTCGACGGGCACACAC
TCACCGTGATCGAGGCGGACGGCACGCTGGTGCAGCCGATGACCGTGCACCGGCTGCCGATTGCGCCCGGGCAG
CGGTACTCGGTGGTGCTGAACCGGGTGCCCACCGCGGACGGCTCGCCGATGCCGCAGCGTGCGTGGATGCGTGC
CGAGATCAGCGACGACTGCTTCCAGTACACGAACCCGGTGCTGGACCTAACCACCCGGGCGATCGTGAGCTACGG
CGAGGAGGAGCGCGACCTGCGGCGGCGGCTGCCGCTCACCACGCGCGGCTCGCCGTTCGACGCGCTCGACACGC
TGCCGACCACGCGCGCGTGGAAGGTGCAGGACCCGGAGGTGCCCGAGGAGCCGTGCCACGACCTGCAGCCGGG
CACGCTCGTGCCGCTGGAGCGGGACCCCGCGCCGGAGCTGCACCTGGACCGCGGCGACCAGCGCATCATGCTCTA
CACCACCGTGGTCGGGCTCAACCGCTGGGACGAGGTGGGCATCAGCTACATGAACCACAGCACCTGGCGGCCGTT
CGGCGGCCCGCACGGCTCGCGCCCGCCGCTGCTGCAGCGCATCGCCCACGGCCGCGGGCTCACGCCGGACGAGT
GGGCCGCGGCCAACGTGACGGACCCCGAGTGGGAGCTGCTCGCCAGCCCGCACCCGAGCAAGCCCGTGGCGGTG
GAGGTGGTGCTGAACAACTACGACGACTCGCCGCATCCCTTCCACCTGCACGGCCACAAGTTCTGGGTGATGGAG
ACACACGAGGCGGACACCGAGTTCGGTGGCCTGGGCGACTGGTCCGACGAGGGGCAGAGTGCACACTACCAGCT
GGACCGCGCCATGAAGCGCGACACGGTGACGGTGCCGATGCACGGCCACGTCGTGCTGCGCTGGGTGGCGGACA
ACCCCGGCGTCTGGGCGTTCCACTGCCACATGGTCGTGCACTTTGCTGCCGGCATGGGCATGGCGTTCGCCGAGA
TGCCCGCCACCCTGCAGGCGTCGCCACCGCCTGCACTGTCCCAATGTGCATAG

>*M. obtusa LAC2*

ATGGATCCGGCGTTCAATACGAAGGCCGCACCTCAAGAACGTTATTTCAACTGGACTATTTCAAGAAATATCCGTG
CACCTGATGGTATTCCCCGCCTGATGTATACTATTAATGACGTTTTCCCAGGCCCGCTTATTGAGGCTAATGAGGGC
GACACGCTGGTAATTCATGTTCGTAACGCTGTTCTTGATAACGAAACACTCCCTGAAGCGCCCATGTCTTCCCAGAT
TGACTCCGTTTTCCCCAAAGGCACTGAGCGCAAACTCGCCATGCACTGGCATGGCCTGTCGATGCGCGATAGCCAA
GTCATGGATGGCGCTGCAGGCTTCTCATCCTGTGTACTTCATCCTGGTGATGAGTACACATATCGATTTAAGC
TGCTTCCCGAGGACGTTGGGACGCACTGGTATCACTCGCATGTTGGCACGTCGCGTGTTGACGGCCTTTG
GGGTATGCTAATTGTACATGCCCGTGAGAATGAAGCTGCCCTCCTACAGCAACATGCAACCAATGTGTCC
GAGTCTCTGAAGTGGGATGAAGAGGTCGCCATCGCACTCGGTGACCACTTCCATGAGCAAGGTCCTGAAT
TCCTTGCCCGCTACGTGTCGCGCTGGATGCAAAAGGCCGAACCTGTTCCTGCAAGTGGCCTTATTAATGG
CAAACATCGCTTCAACTGTGAACACAGCAGGTTAACTCAGGTCCCTTGCCCTGCTGACGAACTTGGTGCG
GATGAGGTGGGAGAATTCTCGACCTTTACCCTGGATCCCAGTAAAAGGTATCGCCTGCGTTTGGTGAATG
TTGGAGCTCTCGCTGAGGAAACGTTCTCGGTGGATGGCCATACACTTACTGTGATTGAAGCAGATGGCGT
CCTTGTCGACCCAATTACAGTGCATCGTATCCCACTCGCAGCTGGCCAGAGATACTCGGTGTTGCTGAAC
CGAGTGAATGAAACTGACTTGGCTTGGATGCGTGCCGAAATGTCGGCTGAATGTTTCCAGTACATGAACC
CCGTTCTGGACCTGGTCACCAAGGCAATCGTCCAGTACACCGAGCCGAGTACAGTCGGTGGCTGGCTCTC
ACCTCTTCGCCGCGCTATGCACGGCCGAGATCTCCTTGCCCACTTCACGTCATCGCAGCGACAGCGTCGA
GCTCTCCAATCCAAGCTGCCCAGCACAACTGGATGGAGCAAGGAAGTGGTTGACCCCGAACTGCCTAGCG
AGCCCTGTCACGACCTGGAATCAGGTGTTCTGGTTCCGCTCATTCCGGATCCAGCTCCAGAGCTGCATCT
AGACCAGGGTGACGTGCGCGAGACAGTACTTATCACCGTCCAGACGCGTGAGCAGTATGGAATCGTGCCG
ATGGGCTTTATGAACCACACGACTTGGCGTGCTGGAGGCGGCGGCGAGCACCCCCGTCCGCCGCTGCTGC
ATCGTATTTCACATTCGAATATGACTGATGCTAGCGCCTGGGCGAAGAATGACCTTGTTAATGACGAGCA
TGAGCTCGTTGTTTCTCCGCATCCAAATCGACCGGTAGTCTTCGAGCTCGTCATCAACAATCATGACGAC
TCCGAGCACCCCTTCCATTTGCACGGCCACAAATTCTGGGTTATGGAAACAGGAGAGGTGGATCCATTCT
GGGGCGGGTACAATGACTACGTGGACCGAGGCCAGACATACGATCTTCGTCGAACTATGAAGCGTGACAC
CTTCGTGATTCCAATGATGGGCCACGCAGTTATCCGCTGGGTTGCGGATAATCCAGGCGTTTGGCCTTTC
CACTGTCATATGTTGGTGCACCTTGAAAGCGGTATGGCCATGGCGATTGCCGAACAGCCCGCTTTGCTCC
AGGCCGCGCCCCCCGTGCCGCCGACTTGCCCCAAGATGTAA

>M. japonica LAC2

ATGAACCCGGCCTATGATACCCAGCAGCCGCCTCAGGAACGAATTTATAACTGGACAATTTCGCGCTCGGAGCGT
GCGCCTGATGGCATTCCTCGTATGATGTACACTATTAATGACCTATTTCCTGGTCCCATGGTAGAGGGAACGGTAG
GCGACACGATGGTTTTCCATGTTCGCAATGCCATCGACGACGATTATAAGATGGAAGAGCCTCCCATGTCATCCCA
AGTCAAAGCAGTGCATCCTAATGGAACCGAGCACCGCATCGCGCTACACTGGCACGGCTTGTCCATGCGGGGTAG
CCAGGTAATGGACGGCGCCCCTGGTTTTACATCGTGCTCGCTCAAGCCCGGCGACGAATTTGTGTACCGCTTTACG
CTGCATCCCGAAGATGTTGGGACACATTGGTACCACTCGCACGTTGGAACATCGCGTGCGGATGGTCTCTGGGGC
ATGTTTACTGTGCATGCTCGCGGCAACGAGCAGGCGGAGCTGGAAGCTCGGGCAAATGACACGTCGCATGCTTTG
CACTGGGATGAAGAGGTGGCGGTCTCGCTTGGCGACCATTTCCACCAGCAGGGCCCTGAATTTCTAGCGTGGTAC
GTCTCTCGCTATTCCCAAAAGGCGGAGCCTGTCCCTGCGAGTGGCCTCATCAACGGCAAGCACCGCTTTAACTGCG
AGCACAGTCGTCTGTCCAATATCCCATGTCCTGCCGACATTTTCGGCAAGGAGGAAGTGGGAGAATACACCACTTT
CACGCTGCAGCCTTCGCGCAGGTACCGCCTCCGTATTGTGAATGTCGGTGCGCTTGCCGACCAGACATTTTCAGTG
GACGGCCATACACTGACGGTCATTGAAGCGGACGGCTTGCTTGTGCAGCCCATCACGGTCCACCGCTTGCCGATT
GCGCCAGGCCAGCGCTACTCGGTCTTGCTGAACCGCGTGAATGACACAGACCGGGCATGGATGCGCTCGGAAAT
GTCGCCCGAGTGCTTCCAGTACCCGAATCCTGTGACAAATTACGAGACGAAGGCCATTGTGTCCTACGCCCCTTCG
GACGAGACTGTGGGCGGATGGCTTTCACCGCTTCGCCGCGCGACGTATGGCCGCGATGTTCTGGCCCGCTTTACTT
CGACGGAGCGCCAGCGCCGCCATGTACAGGCGCTGCTTCCCAACACGGTGGGGTGGGCTAGTGACGTGCAAGAT
CCCGAGGTGCCAACGGAGCCCTGCCACGACCTGGAGCCCGGCACGCTCGTGCCGCTCATCCCTGATCCTGCTCCTG
AACTGCGCCTCGATCAAGGCGATGTGCGCTCGACCGTCTATGTCACTGTTCCTATCCGTGAACGCTGGGGCATTGC
GCCAATGGCGTATATGAACCACACGACGTGGCGCTCAGGCGGCGGTGCCGAATACAAGCGCCCCTCTCTTCTCCA
TCGCATCTCTCACGCAAACTCGACGGATGACAAAGATTGGCGGAAGGATGGCACCGTGGTCGATGAGTACGAGCT
CGTTGTTTCTCCCCACCCCAGCCGCCCTGTGGTGTTTGAATTGGTGATCAACAACCGTGACGACTCCCCTCATCCTTT
TCATCTGCACGGCCACAAGTTCTGGGTAATGGAAACCGGCGAAGTGGACCCCGAATTTGGTGGATTTGACGACTA
CCGCGACGTTGGCCAGACCTATGATTTACAACGTGCTATGAAGCGTGACACCTTCATCGTGCCTATGATGGGCCAT
GCAGTCATCCGCTGGGTCGCGGACAATCCTGGTGTATGGGCGTTCCACTGCCATATGCTTGTCCACCTTGCGAGCG
GCATGGCGATGGCAATTGCCGAGCAGCCGGCCCTTCTCCAAGCCACACCGCCTGTGCCCCCGACCTGTTCATAG

> *LAC2 M. furfur* CBS 14141

ATGGACCCCGCTTTTGCTAGAAAGGCTCCTTCGCAAGAGCGCCTTTACAACTGGACCATATCTAGGGACGTGAGA
GCACCCGATGGAATACCGCGTCTTATGTACACCATCAATGGACTCTTTCCTGGTCCTATAATCGAAGCGAATGAGG
GTGACGTTCTAGCAATCCAGGTACACAACGCCATCTTCGACAACGAAACTCTTCCTAACCCTCCAATGTCTTCCCAG
ATTGATTCAGTGTTTCGCAAGGGCACTGAACGTAGCATCGCTATGCATTGGCATGGTCTCTCCATGCGGGGTACTC
AAGAAATGGATGGTGCAGTTGGATTCTCTTCTTGTGTCCTCCGTCCTGGCGAAACGTTCACTTACCGGTTTGCTTTA
CATGCGGAGGATGTGGGAACCCATTGGTATCATTCACACATTGGTACATCACGAGCTGATGGGCTCTGGGGTATG
CTTATTGTTCATTCCCGACACAACGAAGCGGCTCTTTTACGAAAGTACAGCACAAATGCAACCGAGGCACTTGTGT
GGGACGAAGATGTGGCAGTTGCTTTAGGGGATCATTTCCATGAGCAAGGGCCAGTGTTTTTGGCACGCTATGTTT
CACGGTGGATGCAGAAGGCAGAGCCTGTGCCGGCGAGCGGCCTAATCAACGGAAAGCATCGTTTTAGCTGCGAG
CATAGTCGTCTCACGCAAGTACCATGCCCAGCGGATCCTCTCGGGAAAGAGATTGTCGGGGAGTACTCAACATTTT
CGTTAGACCCTTCAAAACGGTATCGGCTACGTCTAGTCAACGTCGGCTCCCTCGCTGAGCAGACTTTCTCAGTTGAC
GGGCATTCTTTGACTGTAATCGAAGCAGATGGCGTCCTTGTTGATCCATTTACAGTTCATCGGCTTCCAATTGCGAC
AGGACAGCGGTATTCTGTGCTTCTTAACCGTCTTGACGACCAGCAACAAGTATGGATGCGTTCAAAGATGTCTGCC
GAATGTTTTCAGTATATGAACCCTGTTCTGGATTTGGTAACAAAAGCTATTGTGGAATATCAAGACTCAGGACCAA
AGGGAAGCTGGCTTTCACCTCTTCGTCGAGCTGTGAAAGGCCGCAGGGATCTTGCTCAGTTTACATCTTCTCGGCG
TCGGAATCGAGCTTTACAAAGCAAGCTCCCATCCTCGCGCGGTTGGGGCGACAACGTGGAGGATCCCAAAGTTCC
CAGTGAGCCTTGTCATGACCTCGACAACGCAGCGTTGACGCCATTGATTTCAGACCCTGCACCTGAACTAAATTTT
GAACGAGGAGACGTGCGTGAGACTGTGTTAATAACGGTCCAAACACGAGAGAAATATGGAATTGTACCAATGGG
GTACATGAACCACACTACGTGGCGAGCTGGTGGAAGCGGCAAGGTGCCGCGTCCACCTTTGCTTCATCGTATTTCA
CACTCGAATTCCACTGACCCTAATGTATGGGTGGACGATGGGCTAATCGATTATGACCATGAATTAGTGGTTTCTC
CCCATCCCAACAGCTCCGTTGTTATTGAGCTTGTGATCAACAATCATGACGATTCTGAGCACCCGTTTCATTTGCAT
GGGCACAAGTTTTGGGTTATGGAGACAGGCGAAATTGACCCAATGTGGGGTGGCTACAATGACTATGTTGATCGA
GGACAAAGATACGATTTGAACCGCGTTATGAAGCGTGA

>*LAC2 M. furfur* CBS 7982

TCAGTTACAGGCGAACATCTCTAGTGGGCGTGTTTTGCCTTGATGAATCGCCATTTGTTTGTCCCCTGGGTTTTTGC
GGCAGCATTTGTGATAACAGGTCTTCTGGCAGTTGTGTGGTGGATCGTCTCTGATGCGCAACATCAATATTTTCTC
GGAAAACATGCGTTCCCTCTAGTCTCCAGCCGCTGGTTGGAATCTCCGGTCAGCGAAGGTCCTATTGCAATGGACC
CTAATTTTTCCACCAGAGTTCCTCCAAAAGAGCGCCATTACAACTGGACAATATCCAGAGATATGCGAGCACCTGA
TGGAATCCCCCGCCTCATGTACACCATCAATGGTCTTTTTCCCGGACCTCTGATTGAAGCAAATGAAGGTGACACA
CTCGTAATTCGAGTATGGAATGCCATTTTCGACAATGAAACCCTTCCTAGTCCTCCCATGTCATCACAAATTGACTCT
GTGTTTCGAGGGGGTACTGAGAGGAATGTTGCGATGCATTGGCATGGTCTTTCCATGCGGGGCACACAAGAAATG
GATGGAGCAGCTGGGTTCTCTTCATGTATACTCCACCCAGGCGAAATGTTTACTTATCGATTTACCCTACACCCAGA
AGATGTGGGGACCCATTGGTATCATTCACATATCGGGACGTCGCGAGCAGATGGGCTCTGGGGAATGCTGATCGT
TCATTCTCGACACAACGAAGCAAAGCTTTTACAAAAGTACAGCACAAATGTAACCGAGCTCCTTGCTTGGGACGAA
GATGTCGCGATTGCTTTAGGGGATCATTTTCATGAGCAAGGGCCCGTGGTTTTGGCACGTTATGTTTCACGATGGA
TGCAGAAGGCCGAGCCCGTACCTGCCAGTGGTCTGATAAACGGAAGGCACCGCTTCGACTGCGAGCATAGCCGTC
TCACACAAGTGCCTTGCCCAGCAGACCTATTGGGAAAGGATGTTGTCGGTGATTATTCTACATTTTCTTTGGATCCT
GGTAAGCGTTATCGGCTACGTCTAGTCAACGTTGGTTCTCTCGCCGAACAGACGTTTTCGATTGATGGTCATTCCTT
GACAGTCATTGAGGCAGATGGTGTCCTGGTTGATCCATTCACTGTACATCGGCTTCCAATCGCAACAGGTCAGCGA
TATTCTGTGCTTCTTAACCGCCTCGACGACCAGGGTCTAGTATGGATGCGGTCACAAATGTCTGCCGAATGTTTTCA
ATATATGAACCCTGTTTTGAATTTGGTCACAAAGGCTATTGTGGGATACCAAGTCTCGGGACCGAAGGGAAGCTG
GCTTTCACCTCTTCGGCGGGCCATAAAGGGTCGCCAAGAAATTGCTCAATATGCGTCTTCTCAGCTACAGAGGCGT
TTATTTCAAAGCAAGCTACCATCATCTCACGCGTGGGGTGATAATGTGAAAGATCCCAAGATCCCTAATGAACCTT
GCCATGACATTGATACCAAAGACTTAACTCCACTGATTCCTGACCCTGCCCCTGTGCTAGATTTTGGACGGGGCGA
CATACGTGAGACTGTCCTGATAACAGTCCAAACTCGTGAAAAGTATGGAATTGTACCAATGAGCTACATGAACCAT
ACTACATGGCGGGCCGGTGGAAGCGGTAAAGTGCCACGCCCGCCTTTGCTGCATCGTATTTCACACGCAAATTCTT
CCGACCCAACTTCATGGTTAAACGCTGGACTAATTGACCATGATCATGAATTGGTGGTGTCGCCCCATCCTAAAAG
GCCCGTTGTTATTGAGCTTGTGATCAACAATCATGATGATTCTGAACACCCCTTCCATCTACATGGGCATAAATTTT
GGGTCATGGAGACAGGCGAGATTGACCCAGTTTGGGGCGGTTACAATGACTTTGTTGATCGTGGACAAAATTACG
ACTTAAGGCATAAAATGAAGCGTGATACATTTGTTATACCAATGATGGGCTATGCTGTCATTCGTTGGGTAGCCGA
CAATCCTGGTGTGTGGCCATTTCATTGTCATATGCTGGTGCACCTTGAGAGTGGGATGGCTATGGCTATCGCAGAG
CAGCCCGAATTACTTCAGGCAGCTCCTCCAGTACCTCCAGTATGTAG

>*M. yamatoensis FET3*

ATGCTCAAGACACTTCTGAGCCTGGCCTATCTGTCGTTATTTTGTGCTGCTCAAGATATCCAGTACGACTGGAAGCT
AACAAAAGTTCCTAATATCGATCCTTCTGGTCTTTATCCACGTACCGTGCTTGGAATCAATGGGCAATGGCCTCCTC
CCCCTATTAATATCAATTCCACCGATTACTTCAGTATCAAATTCACCAATCAGCTTGGCGATGGTCACCCAAGCGCG
TTGCATTCCCATGGCATGTTCTTCAACCGCACAAACTACTATGATGGCGCTGCAATGATCACGCAATGCCCTATTCC
GGACGGGTCCAGCATGACCTATGATGTGTTGAACTCTCCCAGATCTCCTCCTGAAAGATTCGGTAAGCAATGGGG
CACGTATTGGACGCATTCGCATTACAAGGGCCAGTATGTGGATGGCTTCCGTACCCCGTCGGTGATCCACAATGTG
GATTCTGGCGGAGTGAATCACGAAGCGCATCAGTATGATGATGACTTCACTATCTCTCTCGGTGACTGGTACAATG
AATGGTACGATGTTCTCAATTCGACGGAATTCATGAACGTGAATAATCCTAGTGGTGCTGAGCCGATTCCTGATGC
GCACTTGATTTATTTCCAGCACACACCTTGGAACGGATTCGCTGAGAACCTGCCAGGTTTCAATGAAAATGCCACTT
TGCGTTTCGAAGCGAACAAAACCTATCGTTTGCGTATCGTCAACATGTCGTCTCTGTCCATGTTCTACTTCTGGATC
GAGGGGCATCAAATGGAGATTATCGAAGTGGACGGTGTAGATACCGAAGCTTTCCCAGTGGATTTCATTTCGGTG
TCTGTCGCCCAGCGTTATTCGGTCTTGGTCAAGGCACGCGCCGACGCCGATCCAAAGAACTGGAAGATCAACCTG
GCTGCCGACGGCGATATGTACGACAATGTTCCTGATACTTTGCAAATGAACATTTCTAGTATCATCTCTTATGGCGA
CAATCTTGAAGTCGGCGATGATGGTCGCGCGACGCTTGATGAGTTCGAGGTGTTTGACGACACACAGCTTGTTCCC
GTCGAGCCTATCCCGATGTACGAGTTGTCCGATCCATCCACTCAAAGCCATCGTCTCGATATTTACTTCACCACGTT
TGAAAATGGTATCAACTATGGCACATTCAACAATAATACTTTTGTCGCACCATTGGTCCCTTCGATCATGACGATGA
TGTCGGAAGGCGCAAAGACTTCCGATGCTCGCTACTTTGGTCCAAATTCTCACGCAATTATATCCAAGCATTTGGAT
GTGGTGGAATTTGAATTGTTCAATTGGGATTCCGGTTATCATCCCTTCCATATGCATGGAACCAGTTTCCAGGTGGT
TTGGCGTCAGACGGACCTCACTTCCGACGATCCGGCAGACCATCCAGACTTTAATCCTTTGCAAGAGAACCCTATT
CGCCGCGATACTGTTATGGTACCGCCATCTGGTCGTGTGAAGATTCGCTTCATCGCAGACAACCCAGGAGTCTGGT
TCTTCCATTGTCATATTGACTGGCATCTTGCTTCTGGTTTGGCTCTATTGATGGTTCAAGGTCCAGAAGTATTCCCTT
CCATCTATACGGAGATTCCTACCGACTTTTATCAACAATGTCAGGCTCAAAACTTGAGCACTGCCGGAAATGCAGG
CGGGATTACAGATTCCGTCACAGACTTTGGCGCACTCCCCGAACAGCCGCACTTGCCTCGTGTTGGTTGGACTCCA
GTGATGATCGGCACTTTTGTCGCATGTATCCTAGCTGCTTTAATGGGTATCACTGCATTGTGTTTTTACGGACTTCA
GTCTCACACCGACGAAGACGATGAGGATGAGCACGAACAGGAAAAGTACGAAGACCGCCATGTCGAGTAATGCG
TTGACTAATAG
