# Supplementary material for: Gene Function Analysis in the Ubiquitous Human Commensal and Pathogen Malassezia Genus
Source: mBio. 2016 Nov 29;7(6):e01853-16. doi: 10.1128/mBio.01853-16 (PMC5137500; doi:10.1128/mBio.01853-16)
Supplement: Table S1 — Sequences of oligonucleotide primers used in this study. [file mbo006163076st1.docx]

**Table S1** Sequences of oligonucleotide primers used in this study.

| **Name** | **Sequence (5´- 3´)** | **Purpose (F = forward, R = reverse)** |
| --- | --- | --- |
| ALID2078 | TCCACGGTGCAGATCCTC | p*ACT1* F, and overlap PCR |
| ALID2079 | GTCAAGAGTGGCGGCCGCCATGGTGATGGTGGTGTTACAAGC | p*ACT1* R + *NAT* overlap |
| ALID2080 | CTTTATATGAGTATGCCTTGTCCATAAGTTTTCTAACGATGCGGCATGG | t*ACT1* F + *NAT* overlap |
| ALID2081 | CGTCCTCTCCTATGTCTG | t*ACT1* R, and overlap PCR |
| ALID2139 | AATCCATCTTGTTCAATCATGGTGATGGTGGTGTTACAAGC | p*ACT1* R + *NEO* overlap |
| ALID2140 | CTTGACGAGTTCTTCTGAGTTTTCTAACGATGCGGCATGG | t*ACT1* R + *NEO* overlap |
| ALID2141 | ATGATTGAACAAGATGGATTG | *NEO* F |
| ALID2142 | TCAGAAGAACTCGTCAAG | *NEO* R |
| ai036 | TTATGGACAAGGCATACTCATATAAAG | *NAT* R |
| ai037 | ATGGCGGCCGCCACTCTTGAC | *NAT* F |
| ai76 | AACAGTTGCGCAGCCTGAATG | Inverse PCR |
| ai77 | AGAGGCGGTTTGCGTATTGG | Inverse PCR |
| M13F | GTAAAACGACGGCCAGT | Inverse PCR |
| M13R | CAGGAAACAGCTATGAC | Inverse PCR |
| GI121 | GCGCGCCTAGGCCTCTGCAGGTCGACTCTGTCGATGTATCTGACTTGTC | *LAC2* KO - 5ʹ F |
| GI122 | GAGGATCTGCACCGTGGAGTGTACCTGGATTGCTAG | *LAC2* KO- 5ʹ R |
| GI123 | CAGACATAGGAGAGGACGCAACAGCTCCGTTGTTAT | *LAC2* KO - 3ʹ F |
| GI124 | TGATTACGAATTCTTAATTAAGATATCGAGCTGGAACTGTCTTCCAAG | *LAC2* KO - 3ʹ R |
| GI125 | CGTACACCACCAGTGAATG | *LAC2* KO screening F |
| GI126 | GCATCCTTGTAGAATCGTGC | *LAC2* KO screening R |
| GI139 | GCGCGCCTAGGCCTCTGCAGGTCGACTCTCCAGAATCATGCCACGCAAC | *ADE2* KO - 5ʹ F |
| GI140 | GAGGATCTGCACCGTGGACAATCGCGACAAAGTCCACC | *ADE2* KO - 5ʹ R |
| GI135 | CAGACATAGGAGAGGACGCGAACGACGCACGCGCAC | *ADE2* KO - 3ʹ F |
| GI136 | TGATTACGAATTCTTAATTAAGATATCGAGGTCTCCCAGATAATGATC | *ADE2* KO - 3ʹ R |
| GI137 | ATTCTCCGTACCAATTTC | *ADE2* KO screening |
| GI138 | GCTTCGGCACGTCGTAGC | *ADE2* KO screening |
| GI152 | CTGATCCAAGCTCAAGCTC | Recombination pGI3 F |
| GI153 | GTTGGCCGATTCATTAATGC | Recombination pGI3 R |
| GI154 | GTCGGAGAAGCAGTCAATGC | Recombination pGI3-*NAT* R |
| GI155 | CACCAGGGTTTCCAGTCTC | Recombination pGI3-*NAT* F |
